# Isolate Specific Cold Response of *Yersinia enterocolitica* in Transcriptional, Proteomic, and Membrane Physiological Changes

**DOI:** 10.3389/fmicb.2019.03037

**Published:** 2020-01-23

**Authors:** Chenyang Li, Jayaseelan Murugaiyan, Christian Thomas, Thomas Alter, Carolin Riedel

**Affiliations:** ^1^Institute of Food Safety and Food Hygiene, Department of Veterinary Medicine, Freie Universität Berlin, Berlin, Germany; ^2^Institute for Animal Hygiene and Environmental Health, Department of Veterinary Medicine, Freie Universität Berlin, Berlin, Germany; ^3^Department of Biotechnology, SRM University AP, Amaravati, India; ^4^Department of Food Science and Technology, Beuth University of Applied Sciences Berlin, Berlin, Germany

**Keywords:** *Yersinia enterocolitica*, cold response, proteome, isolates specific, motility, fluidity

## Abstract

*Yersinia enterocolitica*, a zoonotic foodborne pathogen, is able to withstand low temperatures. This psychrotrophic ability allows it to multiply in food stored in refrigerators. However, little is known about the *Y. enterocolitica* cold response. In this study, isolate-specific behavior at 4°C was demonstrated and the cold response was investigated by examining changes in phenotype, gene expression, and the proteome. Altered expression of cold-responsive genes showed that the ability to survive at low temperature depends on the capacity to acclimate and adapt to cold stress. This cold acclimation at the transcriptional level involves the transient induction and effective repression of cold-shock protein (Csp) genes. Moreover, the resumption of expression of genes encoding other non-Csp is essential during prolonged adaptation. Based on proteomic analyses, the predominant functional categories of cold-responsive proteins are associated with protein synthesis, cell membrane structure, and cell motility. In addition, changes in membrane fluidity and motility were shown to be important in the cold response of *Y. enterocolitica*. Isolate-specific differences in the transcription of membrane fluidity- and motility-related genes provided evidence to classify strains within a spectrum of cold response. The combination of different approaches has permitted the systematic description of the *Y. enterocolitica* cold response and gives a better understanding of the physiological processes underlying this phenomenon.

## Introduction

*Yersinia enterocolitica*, the third most commonly reported foodborne zoonotic pathogen in the European Union, can cause serious diseases, including gastroenteritis, mesenteric lymphadenitis, reactive arthritis, erythema nodosum, and pseudoappendicitis (Ostroff et al., 1994; Horisaka et al., 2004; European Food Safety Authority and European Centre for Disease Prevention and Control, 2016). It occurs ubiquitously in the natural environment and is widespread in animal populations (Benembarek, 1994; Robins-Browne, 2013). Furthermore, it can be isolated frequently from a variety of foods, including milk and milk products, pork, poultry, eggs, and produce (Bari et al., 2011).

*Yersinia enterocolitica* is capable of growing at temperatures approaching and even below 0°C (Tudor et al., 2008; Divya and Varadaraj, 2013). Therefore, even refrigeration temperatures (0–4°C) can allow significant bacterial growth over time. Several studies have reported growth of *Y. enterocolitica* in food products stored at refrigeration temperatures: e.g., on raw beef, with increased cell counts of up to 2 log CFU/ml within 4 days (Tudor et al., 2008) and in pasteurized milk, reaching levels of 5–7 log CFU/ml after 7 days (with an initial inoculum of 1–3 log CFU/ml) (Amin and Draughon, 1987).

One of the most prominent cold responses is the induction of cold-shock proteins (Csps) in all psychrotrophs, mesophiles, and thermophiles (Polissi et al., 2003; Phadtare, 2004). As model systems, *Escherichia coli* and *Bacillus subtilis* have been studied in detail regarding cold response and Csps (Phadtare et al., 1999; Ermolenko and Makhatadze, 2002; Weber and Marahiel, 2003). The role of polynucleotide phosphorylase (PNPase, encoded by the *pnp* gene) in regulating cold response is also well described (Goverde et al., 1998; Yamanaka and Inouye, 2001; Cordin et al., 2006; Matos et al., 2009; Phadtare, 2011). This enzyme with the 3’- to 5’-exonucleolytic activities involved mostly in mRNA decay and ribosomes release (Coburn and Mackie, 1998; Polissi et al., 2003) is used to help repress the generation of Csps and relieve growth arrest (Neuhaus et al., 2003; Zhao et al., 2016). Meanwhile, in psychrotrophic bacteria such as *Arthrobacter globiformis* and *Pseudomonas fragi*, some cold-responsive proteins are synthesized at relatively moderate levels and prolonged in response to continuous growth at low temperatures (Berger et al., 1996; Michel et al., 1997). These proteins are of particular importance since they differentiate psychrotrophs from mesophiles, and they are probably one of the key determinants that allow survival at low temperature (Hébraud and Potier, 1999). Additionally, the ability to cope with temperature downshift must be accompanied by a number of changes in response to alterations of physical and biochemical parameters, including solubility, membrane fluidity, protein conformation and stability, and changes in gene expression (Hébraud and Potier, 1999; Vorachek-Warren et al., 2002; Albanesi et al., 2004; Phadtare, 2004; Cao-Hoang et al., 2010; Barria et al., 2013). Therefore, the biochemical and physiological effects allowing bacteria to adapt to temperature changes are likely to be complex, involving a number of cellular processes.

As a psychrotrophic bacterium, *Y. enterocolitica* has two well reported *csp* homolog genes (*cspA* and *cspB*), which are strongly expressed during the cold response. The cold-shock exoribonuclease PNPase and *pnp* gene have also been reported (Goverde et al., 1998; Phadtare, 2011). Additionally, a previous study has reported that genes involved in various functions (regulation, motility, virulence, and metabolism) are upregulated after a temperature downshift from optimal (30°C) to suboptimal (10°C) conditions in *Y. enterocolitica* (Bresolin et al., 2006). However, the effects of these genes and the cold response on protein expressional levels are not clarified in *Y. enterocolitica*.

Recently, advances in proteomics and bioinformatics technologies provide clear information on protein expression in response to cold and other stresses. High-throughput comparative proteomics with label-free quantification enabled the parsing of various potential mechanisms and regulatory networks of stress response in *E. coli*, *B. subtilis*, *Pseudomonas putida*, and *Yersinia ruckeri* (Delumeau et al., 2011; Stefanopoulou et al., 2011; Herbst et al., 2015; Kumar et al., 2016).

However, to our knowledge, the global proteomic profiles of *Y. enterocolitica* under the influence of low temperature have not been reported. Considerable research on *Y. enterocolitica* cold response has been limited to few proteins or genes and to single time points. The aim of this study is to describe the physiological processes of cold response in *Y. enterocolitica* via comparisons of growth ability, expression of cold-responsive genes and proteins, as well as cell motility and membrane fluidity of selected strains upon exposure to cold conditions.

## Materials and Methods

### Growth Profile at Low Temperature

In order to test the growth ability of *Y. enterocolitica* at low temperatures (4°C), 55 isolates were collected from different matrices, representing different serotypes and biotypes (details are given in Table 1). Isolates were incubated on Plate Count agar (PC agar, Merck, Darmstadt, Germany) at 28°C for 24 h. Single colonies were transferred to 3 ml of *Brucella* broth (BB, BD Franklin Lakes, NJ, United States) and incubated at 28°C for 20 h. Enriched cultures were serially diluted 1:10^6^ in BB to reach a cell concentration of about 10^1^–10^2^ CFU/ml as the initial value. Growth abilities of 55 strains were tested based on cell concentration in BB after incubating at 4°C for 168 h. For growth profile investigation, cell concentration of the selected isolates (II7D, 8081, and 44B) was measured under cold stress for 0, 24, 48, 72, 144, and 168 h respectively. The experiment was carried out in six biological replicates (with two technical duplicates each).

**TABLE 1:** Characteristics and growth ability of *Y. enterocolitica* strains at 4°C for 168 h.

**Isolates**	**Median**	**Median norm.**	**Serotype**	**Biotype**	**Matrix**
44B	1.86E + 03	2.86E + 02	O:5,27	1A	Food
IP566/82	4.60E + 03	8.85E + 02	O:8	n. a.	n. a.
4780	5.90E + 05	5.98E + 04	O:8	1B	Human
96/10	1.50E + 05	1.01E + 05	O:8	1B	n. a.
39/91	5.00E + 06	2.87E + 05	O:8	1	Human
21/08	4.20E + 07	3.41E + 06	O:8	1A	n. a.
8081	2.70E + 07	6.85E + 06	O:8	1B	Human
78/90	1.07E + 08	7.96E + 06	O:8	1B	Human
25 Ia	1.25E + 08	1.22E + 07	O:3	4	Food
96B	2.60E + 08	2.23E + 07	O:5,27	3	Animal
177B	2.40E + 08	2.37E + 07	O:5,27	2	Human
207 IIa	3.60E + 08	2.64E + 07	O:9	3	Animal
207 Ia	3.40E + 08	3.56E + 07	O:9	3	Animal
25/13	8.10E + 08	4.60E + 07	O:5	1A	Food
56/14	5.90E + 08	4.66E + 07	O:5	1A	Food
57/14	7.40E + 08	4.76E + 07	O:9	2	Food
54/13	6.45E + 08	4.93E + 07	O:8	1A	Food
28/07	1.00E + 09	5.03E + 07	O:9	3	Animal
04/13	4.90E + 08	5.05E + 07	O:5	1A	Food
32/07	4.30E + 08	5.77E + 07	O:3	4	Animal
05/13	5.80E + 08	5.79E + 07	O:5	1A	Food
44/07	1.14E + 09	5.92E + 07	O:3	4	Food
III15D	7.00E + 08	5.93E + 07	O:5	1A	Food
24/14	4.50E + 08	5.97E + 07	O:5	1A	Food
29/07	8.30E + 08	5.99E + 07	O:9	3	Animal
09/11	1.37E + 09	6.12E + 07	O:9	2	Food
37/12	5.40E + 08	6.18E + 07	O:5	1A	Food
65/14	1.03E + 09	6.29E + 07	O:5	1A	Food
47/13	5.50E + 08	6.31E + 07	O:5	1A	Food
77/14	6.40E + 08	6.46E + 07	O:9	2	Food
I15C	6.80E + 08	6.92E + 07	O:3	3	Animal
20/07	1.42E + 09	7.17E + 07	O:9	3	Human
11/07	1.07E + 09	7.32E + 07	O:3	4	Human
38/12	5.60E + 08	7.35E + 07	O:5	1A	Food
58/07	1.13E + 09	7.73E + 07	O:3	4	Animal
31/13	7.60E + 08	7.86E + 07	O:5,27	2	food
45/14	1.21E + 09	7.97E + 07	O:5,27	2	Food
03/13	7.00E + 08	8.01E + 07	O:8	1A	Food
387/09	8.20E + 08	8.06E + 07	O:9	n. a.	Animal
06/13	4.30E + 08	8.36E + 07	O:8	1B	Food
18/07	1.30E + 09	8.45E + 07	O:9	3	Human
15/12	9.40E + 08	8.92E + 07	O:5,27	2	Food
19/07	1.07E + 09	9.62E + 07	O:3	4	Human
61/07	1.12E + 09	1.00E + 08	O:3	4	Animal
13/14	1.12E + 09	1.01E + 08	O:3	4	Food
12/07	8.80E + 08	1.02E + 08	O:3	4	Human
30/14	9.50E + 08	1.04E + 08	O:5,27	2	Food
33/07	4.50E + 08	1.09E + 08	O:3	4	Animal
25/14	8.80E + 08	1.12E + 08	O:5,27	2	Food
11/09	1.07E + 09	1.14E + 08	O:5,27	2	Food
14/07	1.45E + 09	1.15E + 08	O:3	4	Human
46/14	1.56E + 09	1.21E + 08	O:5,27	2	Food
89/14	1.42E + 09	1.27E + 08	O:3	4	Food
17/07	9.70E + 08	1.27E + 08	O:9	3	Human
II7D	1.02E + 09	1.27E + 08	O:5	1A	Food

*Median norm.: relative median value normalized with the initial concentration, respectively.*

### RNA Extraction Under Cold Stress

*Yersinia enterocolitica* isolates were selected for RNA extraction. Pre-culture was prepared in 12 ml BB at 28°C (as incubation temperature) for 24 h. The suspension was diluted in BB to 0.05 OD_600_ value and then incubated at 28°C for 2 h to reach an OD_600_ value between 0.1 and 0.2. After centrifugation, the bacteria were suspended into 10 ml cooled BB and incubated at 4°C for different time periods (5 min, 30 min, 2 h, 4 h, 24 h, and 48 h). The pellet suspended in BB at room temperature was used as control. Cold-shock stop mix solution (5% Roti-Aqua-phenol, 95% ethanol, Carl Roth, Karlsruhe, Germany) was added and samples were processed as described elsewhere (Blomberg et al., 1990). All samples were frozen at −80°C until further use.

RNA was extracted with Roti-Aqua-Phenol (Carl Roth). RNA quality of samples was tested by gel electrophoresis. The ratio of absorbance *A_260_/A_280_* and *A_260_/A_230_* were used to assess the purity of RNA photometrically with NanoDrop^TM^ 2000/2000c Spectrophotometers (Thermo Fisher Scientific). A ratio of ∼2.0 is generally accepted of *A_260_/A_280_* and the expected *A_260_/A_230_* values are set in the range of 2.0–2.2. Reverse transcription was performed with Maxima H Minus First Strand cDNA Synthesis Kit (Fermentas, St. Leon-Rot, Germany). The cDNA samples were diluted 1: 5 with nuclease-free water for RT-qPCR investigation.

### Expressional Analysis of Cold-Responsive Genes

Real-time quantitative PCR (RT-qPCR) was used to test the transcription level of cold-responsive genes of *Y. enterocolitica*. Eight genes, which were reported to have enhanced at transcriptional levels at 10°C (Bresolin et al., 2006), were tested in this study. These genes cover the functions of regulation, metabolism, and motility (Supplementary Table S2 lists target genes and used primers). The SsoFast EvaGreen Supermix (SYBR-green, Bio-Rad, Munich, Germany) was used for RT-qPCR assays. The expression of the genes was normalized to the reference gene *polA* (DNA polymerase I) (Townsend et al., 2008). The results of RT-qPCR were visualized and evaluated by CFX software (Bio-Rad).

### Whole Cell Protein Extraction

Three isolates (*Y. enterocolitica* strains II7D, 8081, and 44B) were subjected to incubation at 4°C for 0, 5 min, 2 h, and 24 h. The cells were harvested and the pellet was washed with PBS. Cell pellets were reconstituted with 300 μl distilled water and inactivated by addition of 900 μl ethanol. After the centrifugation and evaporation, the final pellet was reconstituted with 250 μl 20 mM HEPES (pH 7.4) and subjected to sonication for 1 min (cycle, 1.0; amplitude, 100%) with a sonicator (UP100H; Hielscher Ultrasound Technology, Teltow, Germany). Supernatants were collected and the concentration was measured using modified Bradford’s method with Coomassie Plus^TM^ Protein Assays (Thermo Fisher Scientific, Rockford, IL, United States) and the samples were stored at -20°C for further analysis. Each strain was tested six times independently.

### *In-Solution* Trypsin Digestion

The *in-solution* trypsin digestion of proteins was performed as described previously (Wareth et al., 2016). Briefly, 10 μg protein was used for acetone precipitation. The resultant peptides were then reconstituted with 20 μl denaturation buffer containing 6 M urea/2 M thiourea in 10 mM HEPES (pH 8.0) and reduced with 10 mM dithiothreitol in 50 mM of ammonium bicarbonate (ABC, Sigma, Germany). The alkylation was carried out with 55 mM iodacemtamide and subsequently 0.5 μg/μl LysC solution was added. The urea concentration was decreased by 0.5 μg/μl trypsin and the trypsin digestion was stopped by 5% acetonitrile/3% trifluoroacetic acid.

### Liquid Chromatography-Electrospray Ionization-Tandem Mass Spectrometry (LC-ESI-MS/MS) Measurements

Liquid chromatography-electrospray ionization-mass spectrometry (LC-ESI-MS/MS) measurements were carried out as described elsewhere (Wareth et al., 2016). Resultant peptides of trypsin digestion were desalted by solid phase extraction and the peptides were separated using Dionex Ultimate 3000 nanoLC (Dionex/Thermo Fisher Scientific, Idstein, Germany) on fritless silica micro-columns with an inner diameter of 100 μm. Mass spectrometry measurements were carried out using LTQ Orbitrap Velos mass spectrometer (Thermo Fisher Scientific, Bremen, Germany). The LTQ-Orbitrap was operated in the positive mode to simultaneously measure full scan MS spectra in the range of *m/z* 300–1700 in the Orbitrap analyzer at a resolution of *R* = 60,000. After that, isolation and fragmentation of the 20 most intense ions in the LTQ part were carried out by collision-induced dissociation.

The raw mass spectra were processed using label-free quantification algorithm of the MaxQuant version 1.3.0.5 (Max Planck Institute of Biochemistry, Martinsried, Germany) (Tyanova et al., 2016) and protein identification was carried out by searching against protein sequence FASTA file of *Y. enterocolitica* strain YE02/02 (Proteome ID: UP000069750, protein count: 4760) with a wide range of homologous strains downloaded from UniProt database. The following parameters were set for protein identification: Initial maximum precursor—7 ppm, fragment mass deviations—0.5 Da; variable modification—methionine oxidation/acetylation of peptide N-termini; fixed modification—carbamidomethylation; enzymes—LysC and trypsin, both with a maximum of two missed cleavages; minimum peptide length—seven amino acids, and target-decoy-based false discovery rate (FDR) for peptide and protein identification—1%.

The statistical analysis was performed using the Perseus software version 1.4.1.3 (Max Planck Institute of Biochemistry, Martinsried, Germany) (Rudolph and Cox, 2019). The LFQ intensities of proteins were imported and transformed to logarithmic scale with base two. The Student’s *t*-test and Benjamini–Hochberg procedure FDR corrections of the significant *p*-values (*p* < 0.05) were applied for identification of differentially expressed proteins.

### Motility Assay

Motility was tested as described for *Y. enterocolitica* (Bresolin et al., 2008). Three strains II7D, 8081, and 44B were assessed by measuring diameters of migration zone at 4°C with motility agar plates (0.3% agar, 0.5% NaCl, and 1% tryptone). Strains were incubated on PC agar plates overnight at 28°C. Single colonies were transferred onto motility agar plates and incubated initially at 37°C for 2 h to start the assay with non-motile bacteria. Plates were subsequently incubated at 28°C (for 21 h) and 4°C (for 44 h).

### Fluidity Assay

Membrane fluidity of *Y. enterocolitica* was measured by a fluorescence polarization or anisotropy value, which corresponds to the reaction to polarized light of a fluorescent probe inside the membrane (Zaritsky et al., 1985; Aricha et al., 2004; Mykytczuk et al., 2007). Briefly, three isolates (*Y. enterocolitica* strains II7D, 8081, and 44B) were prepared and incubated at 4°C for 0, 2, 24, and 48 h with the method described above. Cultured cells were harvested and washed twice with PBS (10 mM, pH 7.4, Merck) and then incubated with 5 μM 1,6-diphenyl-1,3,5-hexatriene (DPH, Sigma–Aldrich, St. Louis, MO, United States) at 37°C for 1 h. Unlabeled cells were used as a scattering reference. The fluorescence polarization was measured using a Cary Eclipse Fluorescence spectrophotometer with Manual Polarizer (Agilent, Santa Clara, CA, United States) at 360 nm excitation and 430 nm emission. Fluorescence anisotropy was calculated by the formula A = [I_VV_ − I_VH_ (I_HV_/I_HH_)]/[I_VV_ + 2I_VH_ (I_HV_/I_HH_)], where I is the corrected fluorescence intensity, and the subscripts V and H indicate the values obtained with vertical or horizontal orientations, respectively. The emission polarized filter was set either in the vertical (I_VV_) or horizontal (I_VH_) position. Decrease in fluorescence anisotropy reflected increases in the fluidity of the lipid bilayer, which controls or alters the mobility of DPH in the membrane.

### Bioinformatics and Statistical Analysis

Cell counts of the growth assays were expressed as the median with range for all the isolates (CFU/ml) and other quantitative data were expressed as the mean with the standard error of the mean. Paired sample *t*-tests were applied to determine differences in growth profile, gene expression, and fluidity. GraphPad Prism 6 was used to carry out the analyses cited above.

The Gene Ontology (GO) database^1^ and the Kyoto Encyclopedia of Genes and Genomes (KEGG) database^2^ were used to classify proteins and related pathways of proteins (Kanehisa et al., 2016). The Clusters of Orthologous Groups (COGs) functional categories of differentially expressed proteins were assigned by BLAST and searched with the COG database^3^ referring to other research (Tatusov et al., 2001; Galperin et al., 2014).

## Results and Discussion

### Growth Profiles of Isolates at Low Temperatures

Altogether, 55 isolates of *Y. enterocolitica* collected from food, humans, and animals were tested for their growth profiles at 4°C after 168 h (end-point analysis). Diverse growth abilities at 4°C among the isolates were observed. Most of the isolates displayed enhanced growth rates at 4°C over 168 h, up to 10^8^ CFU/ml (23.63%) and 10^7^ CFU/ml (61.81%), while a minority of strains (14.6%) showed a slighter increase, up to 10^2^–10^6^ CFU/ml (Table 1). More than 85% of tested strains exhibited enhanced growth rates, which indicated a general survival and growth ability of *Y. enterocolitica* at low temperatures. This result is consistent with the observations of high levels of this bacterium in food products; e.g., meat, milk, cheese, and oysters (Peixotto et al., 1979; Greenwood et al., 1985; Amin and Draughon, 1987; Wang et al., 2009), and natural environmental conditions; e.g., soil and aqueous at low temperature (Asadishad et al., 2013). However, significant differences in growth ability among the tested isolates were observed at 4°C, which demonstrates the growth specificity of isolates at low temperature. Similar observations (specific behavior of strains under low temperature) were found in *Y. enterocolitica* previously. For example, strains with various serotypes survived differently at 4°C in soil and river water (Tashiro et al., 1991). The impact of low temperatures on the survival of *Y. enterocolitica* strains differs when inoculated on raw pork samples at 4 and −20°C for 90 days (Iliev and Najdenski, 2008).

To investigate the cold response of *Y. enterocolitica*, three isolates [44B (1A/O:5,27), 8081 (1B/O:8), and II7D (1A/O:5)] representing low, medium, and high growth ability at 4°C, respectively, were selected for further analysis (Supplementary Table S1).

### Transcriptional Changes of Cold-Responsive Genes at Low Temperature

**FIGURE 1:**
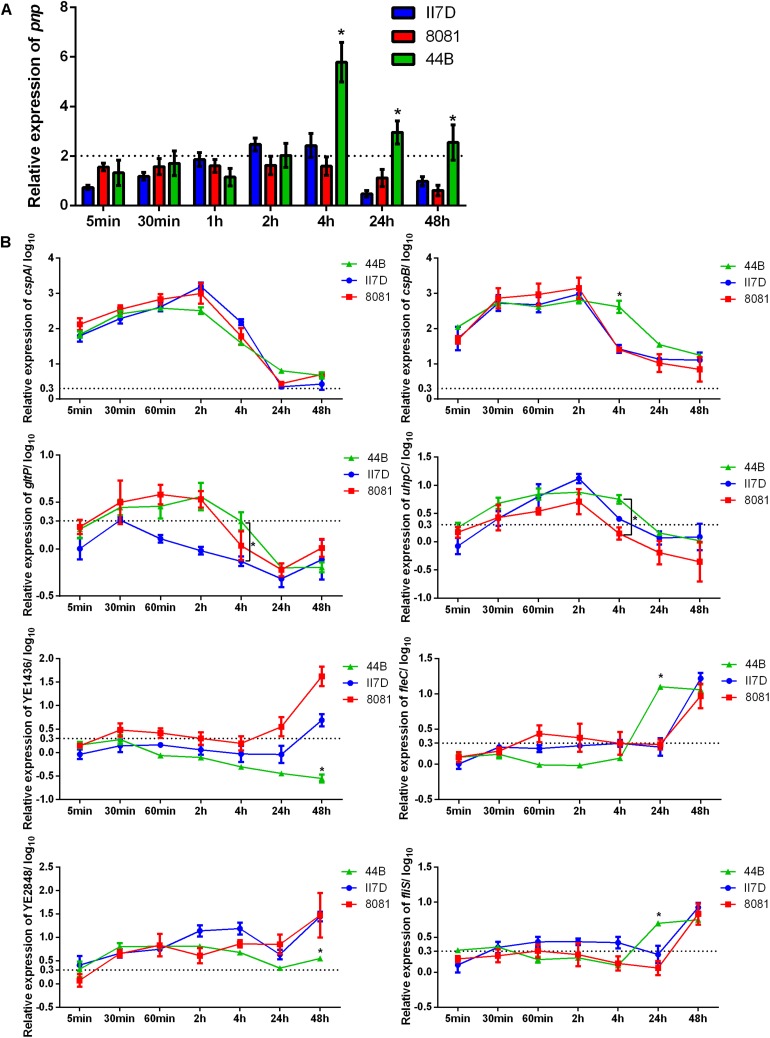
Expressional changes of the cold-responsive genes in II7D, 44B, and 8081. Three *Y. enterocolitica* strains were incubated at 4°C over time (from 5 min to 48 h) to show the expressional changes in cold response. Gene expression was normalized to the reference genes *polA*. **(A)** Expressional changes of *pnp* gene of three isolates at 4°C. Specific values are shown as the means ± SEM of the relative expression in four independent experiments. **(B)** Expressional changes of *cspA*, *cspB*, *gltP*, *uhpC*, YE1436, *fliS*, *fleC*, and YE284 genes at 4°C in three isolates. Specific values of relative gene expression are shown in log_10_ as the means ± SEM of four independent experiments. Statistically significant difference compared with the control according to multiple comparisons (^∗^*p* < 0.05). The line parallel to the *x*-axis represents a biologically relevant induction at 2 (fold-change) and 0.3 (log10 fold-change).

In order to better understand the cold response in *Y. enterocolitica*, the correlation between growth ability and transcriptional changes was investigated in the three isolates. It has been mentioned that *pnp* gene played an indispensable role in the cold response of *Y. enterocolitica* (Goverde et al., 1998) and other bacteria (Mathy et al., 2001; Hu et al., 2014; Briani et al., 2016). In our study, during a cold response, an increased expression of *pnp* gene was detected (Figure 1A). When exposed to 4°C for 5 min to 2 h, the *pnp* expression of the three isolates exhibited no significant difference. After 4 h of exposure at 4°C, the expression of *pnp* in 44B increased continuously and significantly exceeded that of II7D and 8081. The results indicated that different changes of *pnp* expression were found among tested isolates with various growth ability, which verified the essentiality of the *pnp* gene in cold adaptation. The continuous high expression of *pnp* gene implies the higher demand of PNPase and *pnp* in 44B.

Based on the role of PNPase (encoded by the *pnp* gene) in repressing the generation of Csps and relieving growth arrest (Neuhaus et al., 2003; Zhao et al., 2016), the changes in related genes were investigated. RT-qPCR was performed with eight genes, which were reported to have increasing peaks or steady enhancement in gene expression after temperature downshift (Bresolin et al., 2006). The RNA used for this analysis was extracted from isolate cultures kept at 4°C from 5 min to 48 h and the related genes in response to cold with various functions are listed in Supplementary Table S2 accordingly.

As Figure 1B shown, the expression of the genes *cspA*, *cspB*, *gltP*, and *uhpC* increased rapidly after a cold stimulation and then decreased over time, which is consistent with the result from previous study regarding changes of the cold-shock genes (Bresolin et al., 2006; Horn et al., 2007). Based on the expression of these cold-shock genes, the expression decreased rapidly in strains II7D and 8081 after the transcriptional peak. However, in strain 44B, the expression of these genes decreased slowly and the relative expression of *cspB*, *gltP*, and *uhpC* was higher than that of II7D and 8081 at the end of 4 h after cold stress. Since the function of PNPase was RNA degradation and the higher expression of *pnp* was observed in 44B (Figure 1A), the repression of Csp generation might not be accomplished in 44B.

As reported previously, after the repression of Csp production, the growth reinitiated at the end of the acclimation phase (Yamanaka and Inouye, 2001). Therefore, the RNA degradation of Csps by PNPase was indispensable for cold acclimation and growth resumption. Similar cold acclimation was also found in *E. coli*, in which the synthesis of Csps transiently increases and the control of mRNA stability and translatability plays a major role in the adaptive response to cold temperature (Phadtare et al., 1999; Briani et al., 2016).

A different cold response was detected on transcriptional levels of YE1436, *fleC*, *fliS*, and YE2848, which did not show increased peaks but mostly increased under cold stress over prolonged growth. According to the expression of genes YE1436 and YE2848, the transcriptional levels increased over time and the upward tendencies in II7D and 8081 are more obvious than that in 44B (even no obvious uptrend of YE1436 gene expression). After 48 h of cold stress, the relative expression of YE1436 and YE2848 was significantly lower in 44B compared with II7D and 8081. Considering the worse growth ability of 44B at low temperature, the transcriptional regulation of gene YE1436 and YE2848 might be necessary for cold response. As it was mentioned in other studies, one of the psychrotrophic abilities in bacteria was to produce several non-Csps and allow growth during prolonged low temperatures in cold adaptation (Berger et al., 1996; Hébraud and Potier, 1999; Wouters et al., 2000; Phadtare, 2004). After the cold acclimation, the expression of non-cold shock genes has not been resumed in 44B, arresting the transition from acclimation to cell growth.

In addition, after 48 h of cold stress, the expression of *fleC* and *fliS* genes increased in 44B while their expression did not increase until 24 h in II7D and 8081. Since the genes *fleC* and *fliS* are associated with bacterial motility, the regulation of motility might contribute to cold adaptation as well.

Consequently, the transcriptional changes in cold-responsive genes play an important role in both cold acclimation and prolonged adaptation. The isolate-specific ability to survive under cold stress depends on the capacity of enabling transient induction and effective repression of cold-shock gene in cold acclimation. Meanwhile, the resumption of the non-cold shock gene expression was also required in prolonged cold adaptation.

### Global Proteomic Analysis of the Cold-Responsive Proteins at Low Temperature

Three isolates (II7D, 8081, and 44B) with various growth abilities were used for the proteomic analysis to further investigate the underlying processes of cold response. A total of 1526 proteins were identified using label-free quantification analysis in six biological replicates. Among these, 809 proteins which expressed differentially under cold stress (at 4 versus 28°C) for 5 min, 2 h, and 24 h in three strains were identified. Functional classification and annotation indicated that 715 and 790 uniproteins were assigned to 30 GO annotations and 138 KEGG functional pathways, respectively.

**FIGURE 2:**
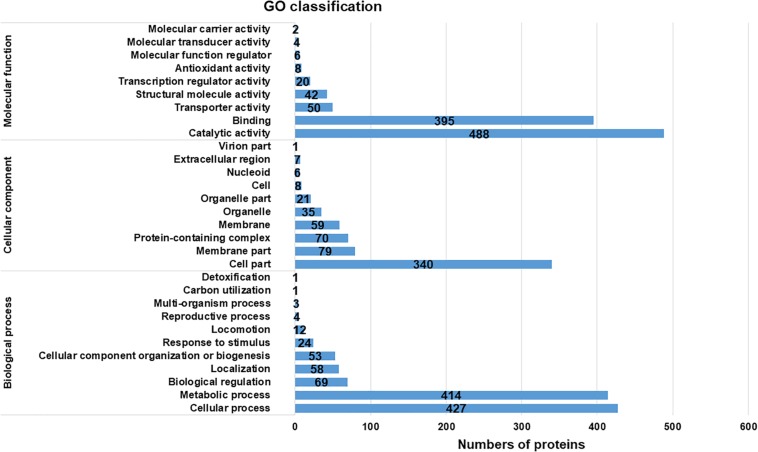
Gene Ontology classification of the total assembled uniproteins.

The proteins assigned to GO functional groups were classified into three categories: “biological process,” “molecular function,” and “cellular component” (Figure 2). Various biological processes were involved in cold response. The most predominant processes were cellular and metabolic process; other major process categories were biological regulation, localization, and cellular component organization or biogenesis. These results indicated that the effects of cold response on protein level in *Y. enterocolitica* were involved in multiple processes. Furthermore, the predominant molecular functions of expressed proteins were associated with catalytic activity and binding; molecular functions of transporter and structural molecule activity were also involved in. Additionally, the most predominant cellular components were located cell and membrane parts. These results implied that the metabolism of the bacteria changed severely after cold response and it might lead to the alterations of cell and membrane components. Considerable groups of temperature-associated proteins were also reported previously in many other studies. For example, the periplasmic proteins associated with cellular component organization are strongly altered in *Yersinia pestis* in response to temperature changes (Pieper et al., 2008). The proteins involved in metabolic processes highly expressed at 4°C in *Listeria monocytogenes* (Cacace et al., 2010). During an abrupt temperature downshift in *E. coli*, expressional alterations occurred in the proteins associated with transport and binding (Kocharunchitt et al., 2014).

**FIGURE 3:**
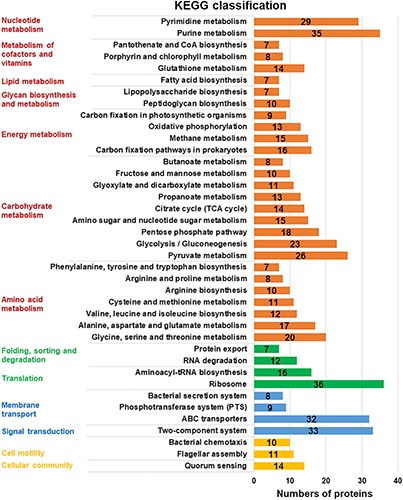
KEGG pathway clusters of assembled uniproteins. Metabolic pathways in different functional groups involved in cold response were classified with KEGG database in four related categories (protein numbers of each group higher than 10): “metabolism” in red, “genetic information processing” in green, “environmental information processing” in blue, and the “cellular processes” in yellow. The subcategory titles were also represented.

For further investigation, the KEGG database was used and the expressed proteins were identified in four categories: “metabolism,” “genetic information processing,” “environmental information processing,” and “cellular processes” (Figure 3). It displayed that “Metabolism” with seven subcategories was the most enriched, which verified the active metabolic changes after a cold response. Among these subcategories, more proteins were enriched in metabolic related pathways: carbohydrate metabolism, nucleotide metabolism, amino acid biosynthesis, and translation. Similar pathways involved in cold response were also described in *L. monocytogenes* and *E. coli* (Cacace et al., 2010; Kocharunchitt et al., 2014).

### Analysis of Differentially Expressed Proteins at Low Temperature

**FIGURE 4:**
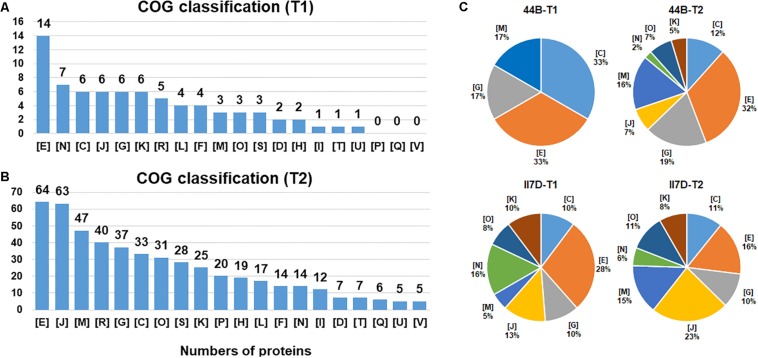
Functional category distribution of proteins identified from *Y. enterocolitica* cells at low temperature. **(A)** The number of identified proteins belong to each functional category is shown under cold stress after 2 h (T1). **(B)** The number of identified proteins belong to each functional category is shown under cold stress after 24 h (T2). **(C)** The percentage of differential expressed proteins that belong to each functional category in II7D and 44B is shown under cold stress in two time (T1 and T2). COG categories are as follows: C: energy production and conversion; D: cell division and chromosome partitioning; E: amino acid transport and metabolism; F: nucleotide transport and metabolism; G: carbohydrate transport and metabolism; H: coenzyme metabolism; I: lipid metabolism; J: translation, ribosomal structure and biogenesis, K: transcription; L: DNA replication, recombination and repair; M: cell wall/membrane/envelope biogenesis; N: cell motility; O: post translational modification, protein turnover, chaperones; P: inorganic ion transport and metabolism; Q: secondary metabolites biosynthesis, transport and catabolism; R: general function prediction only; S: function unknown; T: signal transduction mechanisms; U: intracellular trafficking, secretion, and vesicular transport; V: defense mechanisms.

To investigate the alteration of metabolism related to growth profile under cold temperature over time, differentially expressed proteins were investigated at different time points in two isolates, 44B and II7D (with low and high growth ability at 4°C). Differentially expressed proteins of 44B and II7D under cold stress for 2 h (early stage, T1) and 24 h (late stage, T2) were compared (Figure 4).

Differentially expressed proteins were classified into 20 COGs functional groups with a relative fold change [log_2_ (FC) > 1.2 and log_2_ (FC) < -0.8, *p* < 0.05]. The expressed protein response to the early stage of the cold response (T1) was enriched into 17 functional clusters. Of these, the most predominant categories were “amino acid transport and metabolism,” “translation, ribosomal structure, and biogenesis,” “carbohydrate transport and metabolism,” “cell motility,” and also “transcription.” For the late stage of the cold response (T2), a high abundance of proteins was observed for categories of “amino acid transport and metabolism,” “translation, ribosomal structure, and biogenesis,” “cell wall/membrane/envelope biogenesis,” and “carbohydrate transport and metabolism.” In addition, a high abundance of the proteins belonging to “general function prediction only” was also found in both stages (Figures 4A,B). Throughout the whole testing course (T1–T2), proteins in specific functions of amino acid transport and metabolism (E), translation, ribosomal structure and biogenesis (J), carbohydrate transport and metabolism (G), and energy production and conversion (C) had higher enrichment in both T1 and T2. Hence, the proteins are involved mostly in metabolism in response to cold. Similarly, the high abundances of proteins regarding metabolism-related pathways and metabolic process were also investigated in the KEGG and GO analysis. Hence, we assume that the major cold-responsive proteins participate in the metabolic regulation of cells.

However, differences in protein abundance were observed between T1 and T2. Especially, protein abundance existed mostly in the clusters of cell motility (N) and transcription (K) in T1 while cell wall/membrane/envelope biogenesis (M) and post-translational modification, protein turnover, chaperones (O) in T2. This result indicates that the effects of the cold response on protein levels differ in the early and late stages. The time-dependent differences in protein categories were also found in *E. coli* in response to temperature and water-activity changes and were closely related to the cultivability after the temperature downshift (King et al., 2016). Different phases including adaptation and re-growth phases could be divided based on clustering analyses. Additionally, various protein categories were involved such as energy metabolism, DNA repair system, amino acid biosynthetic pathways, and carbohydrate catabolism (King et al., 2016).

According to the COG classification, eight protein clusters with the most protein abundance in T1 or T2 were chosen to compare the differences between strain 44B and II7D (Figure 4C). Compared with the protein abundance in the other three pie charts, protein clusters of (K), (O), (J), and (N) were undetectable in the early stage of 44B (T1) and the proportions of these proteins in all selected proteins in 44B (T2) were lower than those in II7D (T1) and II7D (T2). This result demonstrates that the biogenesis of responding proteins in 44B lags behind II7D under cold stress. Meanwhile, the proteins in clusters of (K), (O), and (J) represent key processes of protein biosynthesis. Hence, lower abundances of these proteins in 44B (T1) and 44B (T2) suggested that synthesis of general proteins might be inhibited in 44B compared to strain II7D. As was shown in many bacteria (e.g., *E. coli*), the arrest of cell growth upon temperature downshift is caused by the severe inhibition of general protein synthesis (Phadtare, 2004). The inhibition of general proteins in 44B (both in T1 and T2) might be the reason for low growth ability at low temperature. Considering the expressional repression of cold acclimation genes in 44B (Figure 1B), the inhibition might be involved in synthesis of cold acclimation proteins, which are essential for cold adaptation during prolonged growth.

In addition, a lower abundance of protein cluster (N) related to the “cell motility” was also mentioned in 44B. Base on the proteomic results, some cold-responsive proteins related to flagella and chemotaxis were detected in II7D but not in 44B (data not shown). For example, the Flg family, used for flagellar assembly and motility, are temperature-dependent in *E. coli* and other bacteria (Phadtare, 2012; Osterman et al., 2015). The chemotaxis protein, Che family is essential for motility and cold response (Burkart et al., 1998; Liu et al., 2014). According to the transcriptional analysis in Figure 1B, the correlation between motility and growth ability was demonstrated due to the different expressional changes of motility-related genes *fleC* (homologous to *fliC* and encoding Flagellin), *fliS* (putative cytoplasmic chaperone), and YE2848 (putative chemotaxis methyl-accepting transducer) in three isolates. Meanwhile, the Flagellin was detectable only in II7D but not in 44B in proteomic analysis (other related genes were not found). Since it is critical in motility and cold response in *Salmonella enterica* (Elhadad et al., 2015; Michaux et al., 2017), the involvement of motility in cold response might be confirmed.

On the other hand, the percentages of clusters of energy production and conversion (C), carbohydrate transport and metabolism (G), and cell wall/membrane/envelope biogenesis (M) in 44B (T1) are higher than those in 44B (T2), II7D (T1), and II7D (T2). Considering the high abundance of proteins related to carbohydrate metabolism and cell wall/membrane/envelope biogenesis, but low enrichment of proteins related to functional protein synthesis in strain 44B (T1), we might assume that 44B uses a high rate of energy for the cell wall structure, instead of initial growth at cold response. As an important protective structure against adverse environmental conditions, the cell membrane plays an important role in stress response. Previously, it was extensively discussed that membrane lipopolysaccharide, cell membrane, and the membrane fluidity contribute to temperature adaptation in bacteria (Carty et al., 1999; Phadtare, 2004; Storz and Hengge, 2010).

### Motility at Low Temperature

**FIGURE 5:**
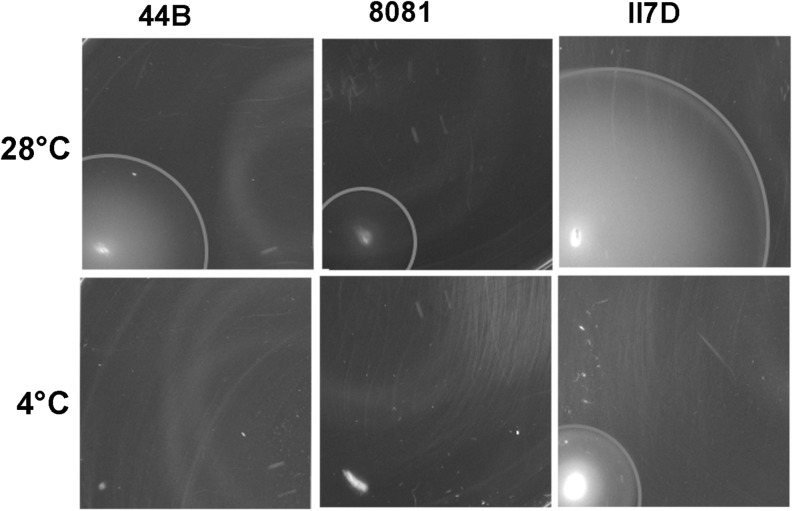
Motility of *Y. enterocolitica* strains at low temperature. Motility was tested on motility agar plate. Single colonies were stabbed on to motility agar plates and incubated initially for 2 h at 37°C to start the assay with non-motile bacteria. The plates were subsequently incubated at 28°C (for 21 h) and 4°C (for 44 h). The motility was assessed by measuring the diameters of migration zone of strains. Values of the migration zone diameters (means ± SEM) in each strains were tested of six independent experiments: 24.92 ± 0.239 cm (44B at 28°C), 11.58 ± 0.201 cm (8081 at 28°C), 49.00 ± 0.966 cm (II7D at 28°C), and 17.83 ± 0.105 cm (II7D at 4°C). No migration zone was detectable in 44B or 8081 at 4°C.

To investigate the physiological changes in cold response, motility assays at low temperatures were performed on three isolates (44B, 8081, and II7D) with different growth profiles at low temperatures. All three strains showed motility at 28°C; however, at the temperature of 4°C, only II7D was motile (Figure 5).

As shown in our transcriptional analysis, the expression of motility-related genes (*fliS* and *fleC*) increased under cold stress at 4°C and their expression was increased earlier in strain 44B than in the other two strains (Figure 1B). Meanwhile, a lower abundance of proteins was present in the “cell motility” group in 44B, which was consistent with the lower growth ability in 44B than II7D. Moreover, the differential growth ability correlates with motility in the three strains (only the strain with high growth ability was motile) at low temperature. Based on the results from transcriptional and proteomic analysis, the different induction of the motility-related genes and proteins among isolates with different growth behaviors indicated the close link between cell motility and growth ability, which has been described previously in *Y. enterocolitica* (Kapatral et al., 1996). However, due to the wide range of factors with complex mechanisms in regulating motility, how cell motility was affected by or contributed to the growth ability after cold response remains unclear (Young et al., 1999; Mukherjee et al., 2013; Xu et al., 2014).

### Cell Membrane and Fluidity at Low Temperature

**FIGURE 6:**
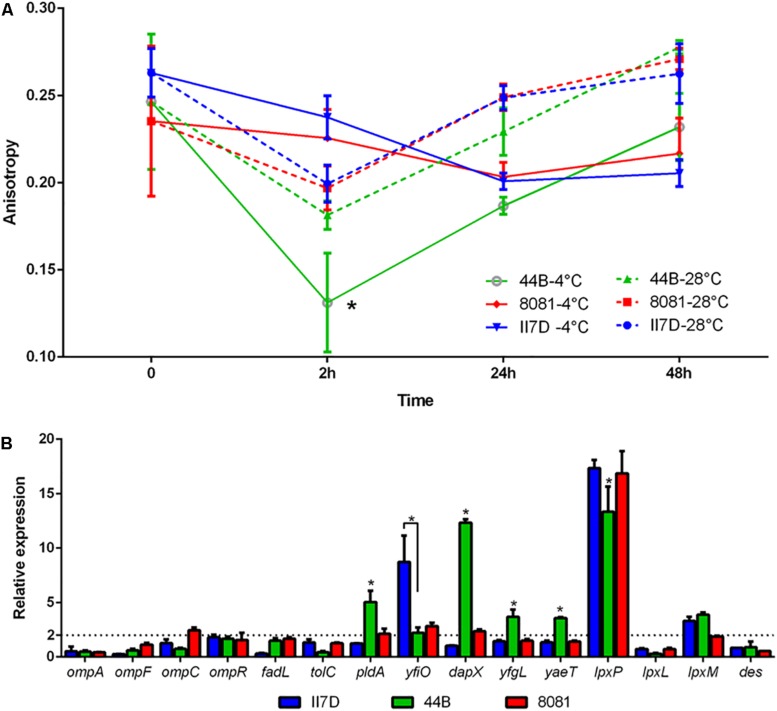
Membrane fluidity and transcriptional changes of membrane-related genes in *Y. enterocolitica* strains at low temperature. **(A)** Fluidity assays in II7D, 44B, and 8081 under cold stress for 2, 24, and 48 h. Anisotropy value represented the membrane fluidity (higher anisotropy means lower fluidity). **(B)** Expressional changes of the membrane related genes were detected using RT-qPCR and were normalized to the reference gene *polA*. Specific values of relative gene expression are shown as the means ± SEM of four independent experiments. The line parallel to the *x*-axis represents a biologically relevant induction at 2 (fold-change). Statistically significant difference compared with the control according to multiple comparisons (^∗^*p* < 0.05).

To test other factors corresponding to membrane activity in cold response, fluidity assays were performed at 4°C on three isolates (44B, 8081, and II7D). All three tested strains showed stable fluidity under the temperature of 28°C in 48 h, while the membrane fluidity of 44B increased significantly at 4°C at 2 h and decreased to the normal level at 4°C after 24 h (Figure 6A). According to the results of fluidity, the membrane fluidity maintained at the normal level in both strains 8081 and II7D, but not in 44B. These results indicated that the balance of the membrane fluidity was changed in response to cold stress in 44B at 2 h. This finding might be correlated to the high protein abundance in the functional cluster of cell wall/membrane/envelope biogenesis in 44B at T1. Therefore, the differences in growth abilities at low temperature might be related to the maintenance of cell fluidity. The similar roles of membrane fluidity have been demonstrated in cold and other stresses in many bacteria (Yoon et al., 2015; Eberlein et al., 2018). However, the fluidities are regulated by various mechanisms in different bacteria; e.g., *E. coli* (Carty et al., 1999), *B. subtilis* (Aguilar et al., 2001), and *Salmonella* (Wollenweber et al., 1983; Ricke et al., 2018).

To investigate the regulatory factors of membrane fluidity in *Y. enterocolitica*, groups of genes regarding outer membrane proteins and lipid A biosynthesis were selected according to previous studies (Dekker, 2000; Nikaido, 2003; Barria et al., 2013; Hussain and Bernstein, 2018; Robinson, 2019). Transcriptional changes in these genes were investigated under cold stress for 2 h in three isolates with the primers listed in Supplementary Table S3. In strain 44B, significantly higher expression of *yaeT*, *yfgL*, *dapX*, and *pldA* and lower expression of *yfiO* and *lpxP* was observed compared to the other isolates (Figure 6B). The four outer membrane protein assembly factors (encoded by *yaeT*, *yfgL*, *dapX*, and *yfiO*) were found in proteomic analysis, in which, BamC encoded by *dapX* was upregulated significantly in 44B. The differential expression of these genes and proteins might be involved in fluidity regulation in response to cold. Similar functions of the outer membrane protein YaeT, DapX, and YfgL (homologous to insert β-barrel proteins in *E. coli*) were shown in previous research in response to cold (Macintyre and Henning, 1990; Onufryk et al., 2005; Wu et al., 2005; Begic and Worobec, 2006; Sklar et al., 2007; Rollauer et al., 2015). Outer membrane phospholipase A (encoded by *pldA*), which is activated under various stress conditions, presents in the outer membrane of Gram-negative bacteria. Its possible role is maintaining the cell envelope integrity and permeabilization, which is related to temperature (Dekker, 2000; Belosludtsev et al., 2014). The different expressions of *pldA* gene and protein among isolates suggested the possible involvement of outer membrane phospholipase A in fluidity maintenance under cold stress. However, their functions in cold response still remain to be elucidated in *Y. enterocolitica*.

Des and LpxP (encoded by *des* and *lpxP* genes) are two fluidity-generated enzymes in *B. subtilis* and *E. coli*. In *B. subtilis*, upon a drop in temperature, the Des protein is synthesized and desaturates the acyl chains of membrane phospholipids to increase the membrane fluidity (Aguilar et al., 2001; Albanesi et al., 2004). Furthermore, in *E. coli*, cold-induced acyltransferase LpxP helps to attach more unsaturated fatty acids (palmitoleate instead of laurate attached at normal temperature by LpxL) to lipid A, thus increasing membrane fluidity and lowering its phase transition temperature, counteracting the effect of low temperature (Vorachek-Warren et al., 2002). In our study, no significant difference in the expression of *des* gene was observed among the three isolates and the Des protein did not induced, which implied that the regulation of Des in *Y. enterocolitica* might not be as important as that in *B. subtilis.* Meanwhile, expression of the cold induced gene *lpxP* was significantly lower in 44B and the related protein were induced differently between II7D and 44B according to proteomic analysis (Supplementary Table S4). Therefore, membrane fluidity related to growth ability in *Y. enterocolitica* might be regulated by LpxP in cold adaptation, which is identical to *E. coli*.

### Proteomic Overview of the Cold Response in *Y. enterocolitica*

**FIGURE 7:**
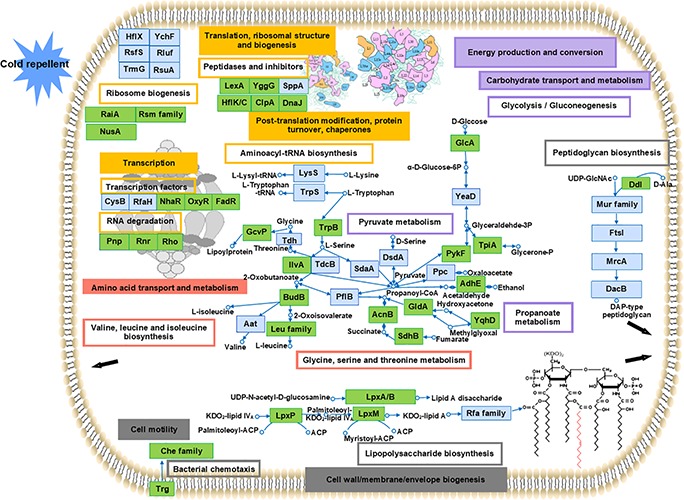
Overview of the cold response of *Y. enterocolitica* at proteomic level. Proteins were selected in the main COG functional categories and distinguished. Boxes with colored border represented the related pathways (including KEGG BRITE hierarchies). The main KEGG pathways reported in each category were presented: transcription factors, RNA degradation, ribosome biogenesis, peptidases and inhibitor, and aminoacyl-tRNA biosynthesis (in yellow); glycine, serine, and threonine metabolism and valine, leucine, and isoleucine biosynthesis (in red); lipopolysaccharide biosynthesis, peptidoglycan biosynthesis, and bacterial chemotaxis (in gray), glycolysis/gluconeogenesis, pyruvate metabolism, and propanoate metabolism (in purple). The related COG categories were marked with the same colors in boxes accordingly. The proteins were displayed in blue boxes representing the involvement in the related functional categories and pathways in this study. The green boxes were used for the proteins also clarified in cold response in other researches. Detailed information is listed in Supplementary Table S4.

Cold response involved a series of complex and significant changes in the abundance of proteins in many processes and pathways rather than a simple increase or decrease in a specific category. To present an overview of the cold response of *Y. enterocolitica*, the upregulated proteins under cold stress were selected according to the main COG functional categories mentioned in this study. These particular proteins probably represented the key determinants that allow life at low temperature. Top KEGG pathways (including the BRITE hierarchies) were selected according to the proteins in COG categories (listed in Supplementary Table S4). Proteomic overview and the predicted regulation in cold response are presented in Figure 7.

First, a high abundance of proteins was observed associated with protein biosynthetic processes, such as transcriptional, translational, and ribosomal, and post-translational processes (related COG categories are shown in yellow boxes). These proteins were involved predominate in transcription factors, RNA degradation, peptidases and inhibitors, ribosome biogenesis, and aminoacyl-tRNA biosynthesis. Numbers of proteins related ribosome biogenesis [such as the transcription termination/anti-termination protein NusA, ribosome-associated inhibitor A (encoded by *raiA*), and ribosomal RNA small subunit methyltransferase B (encoded by *rsmB*)] were induced in response to cold. These related protein associated with cold stress was also reported in *E. coli* and other bacteria (Burakovsky et al., 2012; Di Pietro et al., 2013). Meanwhile, functions of the proteins induced in this study like ribosomal silencing factor RsfS, GTPase HflX, and ribosome-binding ATPase YchF under cold stress have not been clarified previously. Since some of them are involved in other stress response like heat, oxidative, and nutrient stress (Starosta et al., 2014; Hannemann et al., 2016; Dey et al., 2018), their potential roles under cold stress should be investigated. The proteins associated with peptidases and inhibitor such as LexA repressor, lipoprotein (encoded by *yggG*), and Protein HflK were induced under cold stress in this study. The cold-responsive function of these proteins was also found previously in other research (Phadtare and Inouye, 2004; Burakovsky et al., 2012; Jian et al., 2015). Besides, some transcription factors (encoded by *nhaR*, *oxyR*, *fadR*, *cysB*, and *rfaH*) were also involved in regulation of cold response. Since the essential cold-responsive roles of these transcription factors (encoded by *nhaR*, *oxyR*, and *fadR)* were investigated in *E. coli* (White-Ziegler et al., 2008), *Vibrio vulnificus* (Limthammahisorn et al., 2008), and *Moraxella catarrhalis* (Spaniol et al., 2013), transcription factors should also be focused on in *Y. enterocolitica* cold response.

A number of proteins involved in specific amino acids biosynthesis may reflect their importance in mediating survival under cold stress. In our research, the induced proteins participated in biosynthesis of various amino acids under cold stress (related COG categories shown in red boxes). These proteins are associated with the biosynthesis and metabolism of glycine, serine, and threonine (encoded by *trpB*, *gcvP*, *ilvA*, *tdh*, etc.), and valine, leucine, and isoleucine (encoded by *leuA*, *leuB*, *leuC*, *leuD*, *budB*, etc.). Similar amino acids have been demonstrated in previous studies to aid tolerance under cold stress conditions in many other bacteria (Fonseca et al., 2011; King et al., 2016).

Second, proteins associated with cell membrane and motility were identified (related COG categories shown in gray boxes). Certain proteins were identified in pathways, such as lipopolysaccharide biosynthesis (encoded by *lpxA/B/P/M* and *rfaC/Q*), peptidoglycan biosynthesis (encoded by *murC/E*, *ddl*, *dacB*, etc.), and bacterial chemotaxis (encoded by *trg* and *cheB/D/Z*). The cold-responsive functions have been reported previously in many proteins mentioned in Supplementary Table S4. However, although the genes related to motility (*fliS* and YE2848) were detected in our transcriptional, the induction of them cannot be detected in our proteomic results. According to the proteomic data, almost all the proteins related to flagellar assembly were downregulated. The cold-responsive effect of flagella on cell motility at the protein level is unknown.

Meanwhile, valine, leucine, and isoleucine, as the branched-chain amino acids and the precursors for biosynthesis of iso- and anteiso-branched-chain fatty acids, were utilized to regulate the membrane fluidity in response to cold in certain bacteria (Grau and de Mendoza, 1993; Annous et al., 1997; Klein et al., 1999). Levels of isoleucine and leucine significantly increase under cold stress in *E. coli* (Jozefczuk et al., 2010), and the expression of related genes (*leuA/B/C/D* and *ilvB/C/D/E/H*) was also elevated in *Thermoanaerobacter tengcongensis* (Liu et al., 2014). Based on our proteomic results, the induction of l*euA/B/C/D* encoding proteins was only detected in 44B, which implied the indispensable regulation of these branched-chain amino acids. However, the growth ability under cold stress of 44B was detected worse than II7D, which suggested that multiple pathways related to motility might be applied in cold response.

The considerable involvement of proteins has been detected and the transcriptional and physiological investigation associated with motility and fluidity contributes to our understanding of cold-response regulation of motility and membrane fluidity.

Additionally, certain pathways in energy production and conversion and carbohydrate transport and metabolism were also involved in this study (listed in Supplementary Table S4). The complex processes and pathways in cold response of *Y. enterocolitica* and the specific functions of other individual proteins predicted in the proteomic results are required to be investigated during cold adaptation.

This study demonstrates the strain-specific cold response of *Y. enterocolitica* at 4°C, which is time-dependent, including cold acclimation and adaptation. The transcriptional analysis revealed the importance of the induction and repression of cold-shock genes in cold acclimation as well as the resumption of the non-cold shock genes in prolonged cold adaptation. Meanwhile, the time-dependent response at protein level was also found and the cold-responsive proteins identified in proteomic analysis were closely related to protein synthesis, cell membrane parts and cell motility. Additionally, the physiological processes in cell fluidity and motility might be responsible for differential growth abilities at low temperatures. By combining different approaches, cold response was described systematically, providing a better understanding of the significant physiological processes involved in cold stress of *Y. enterocolitica.*

## Data Availability Statement

All datasets generated for this study are included in the article and Supplementary Material.

## Author Contributions

CL contributed to designing, carrying out the experiment, and writing the manuscript. JM provided assistance for the proteome experiment and reviewed the manuscript. CT provided assistance for the experiment. TA reviewed the manuscript and gave advice. CR reviewed the manuscript.

## Conflict of Interest

The authors declare that the research was conducted in the absence of any commercial or financial relationships that could be construed as a potential conflict of interest.

## Footnotes

1 http://www.geneontology.org

2 http://www.genome.jp/kegg/pathway.html

3 http://www.ncbi.nlm.nih.gov/COG/
