# Supplementary material for: Isolate Specific Cold Response of Yersinia enterocolitica in Transcriptional, Proteomic, and Membrane Physiological Changes
Source: Front Microbiol. 2020 Jan 23;10:3037. doi: 10.3389/fmicb.2019.03037 (PMC6990146; doi:10.3389/fmicb.2019.03037)
Supplement: Supplementary file 1 [file Table_1.DOCX]

**S1-1. Growth profiles of *Y. enterocolitica* isolates at 4 °C (CFU/ml)**

| **Time** | **II7D (O:5)** | | | **8081 (O:8)** | | | **44B (O:5,27)** | | | **(p<0.05)** |
| --- | --- | --- | --- | --- | --- | --- | --- | --- | --- | --- |
| **(h)** | **Median** | **Upper limit** | **Lower limit** | **Median** | **Upper limit** | **Lower limit** | **Median** | **Upper limit** | **Lower limit** |  |
| 0 | 8.30E+02 | 1.04E+03 | 6.20E+02 | 4.30E+02 | 5.40E+02 | 3.60E+02 | 6.40E+02 | 1.08E+03 | 5.00E+02 | no |
| 24 | 3.70E+03 | 6.20E+03 | 3.20E+03 | 1.38E+03 | 3.00E+03 | 1.12E+03 | 1.39E+03 | 1.78E+03 | 1.14E+03 | no |
| 48 | 4.30E+04 | 6.00E+04 | 3.20E+04 | 1.05E+04 | 1.38E+04 | 6.00E+03 | 1.82E+03 | 2.60E+03 | 1.60E+03 | no |
| 72 | 5.40E+05 | 8.80E+05 | 4.00E+05 | 5.60E+04 | 8.80E+04 | 4.40E+04 | 2.17E+03 | 3.20E+03 | 1.60E+03 | no |
| 144 | 5.60E+08 | 9.80E+08 | 3.40E+08 | 5.40E+06 | 1.16E+07 | 3.20E+06 | 2.16E+03 | 3.40E+03 | 1.54E+03 | * |
| 168 | 1.02E+09 | 1.12E+09 | 8.60E+08 | 2.70E+07 | 3.80E+07 | 1.40E+07 | 1.86E+03 | 2.30E+03 | 1.74E+03 | * |

**S1-2. Growth profiles of *Y. enterocolitica* isolates at 28 °C (CFU/ml)**

| **Time** | **II7D (O:5)** | | | **8081 (O:8)** | | | **44B (O:5,27)** | | | **(p<0.05)** |
| --- | --- | --- | --- | --- | --- | --- | --- | --- | --- | --- |
| **(h)** | **Median** | **Upper limit** | **Lower limit** | **Median** | **Upper limit** | **Lower limit** | **Median** | **Upper limit** | **Lower limit** |  |
| 0 | 4.70E+02 | 5.60E+02 | 2.40E+02 | 9.60E+02 | 1.02E+03 | 9.20E+02 | 4.30E+02 | 5.80E+02 | 3.80E+02 | no |
| 2 | 1.20E+03 | 1.60E+03 | 1.00E+03 | 3.60E+03 | 4.80E+03 | 1.80E+03 | 9.40E+02 | 9.80E+02 | 6.20E+02 | no |
| 4 | 8.80E+03 | 1.12E+04 | 6.40E+03 | 1.70E+04 | 2.36E+04 | 1.44E+04 | 4.10E+03 | 4.80E+03 | 3.20E+03 | no |
| 6 | 5.90E+04 | 7.20E+04 | 4.40E+04 | 1.79E+05 | 3.06E+05 | 1.44E+05 | 3.20E+04 | 3.80E+04 | 1.80E+04 | no |
| 8 | 3.40E+05 | 4.60E+05 | 3.00E+05 | 2.13E+06 | 2.72E+06 | 1.76E+06 | 1.78E+05 | 2.36E+05 | 1.42E+05 | no |
| 24 | 6.40E+08 | 7.40E+08 | 5.40E+08 | 7.10E+08 | 7.80E+08 | 6.30E+08 | 6.55E+08 | 6.60E+08 | 5.80E+08 | no |
| 48 | 8.00E+08 | 9.00E+08 | 7.00E+08 | 8.40E+08 | 9.80E+08 | 7.60E+08 | 8.90E+08 | 9.60E+08 | 5.60E+08 | no |
