# Supplementary material for: Isolate Specific Cold Response of Yersinia enterocolitica in Transcriptional, Proteomic, and Membrane Physiological Changes
Source: Front Microbiol. 2020 Jan 23;10:3037. doi: 10.3389/fmicb.2019.03037 (PMC6990146; doi:10.3389/fmicb.2019.03037)
Supplement: Supplementary file 2 [file Table_2.docx]

**S2. List of genes selected for the cold-response analysis (based on Bresolin et al., 2006)** (Bresolin et al. 2006).

| **Target gene** | **Primer name** | **5’ – 3’ sequence** | **Function** |
| --- | --- | --- | --- |
| *cspA*, Cold-shock Protein A | CspA F | TTGGTTTCATTACGCCTGCT | Regulation |
|  | CspA R | CCTTTAGCGCCATTCTCGAT |  |
| *cspB*, Cold-shock Protein B | CspB F | CAGACGGCAGCAAAGATGTA | Regulation |
|  | CspB R | GTTTACAGCTGAAGGGCCTT |  |
| *gltP*, Glutamate-aspartate symport protein | GltP F | GTTCCGGGTGTCTCTTTTGT | Metabolism |
|  | GltP R | ACGCGCCATATCCAGAATAC |  |
| *uhpC*, Hexose phosphate transport system regulator protein | UhpC F | AGCATGGCGGGCATAATAAA | Regulation |
|  | UhpC R | GACGGAACAAGTGCAAATCG |  |
| YE1436, Putative transcription regulatory protein | YE1436 F | AAACGATAACAACCCGCTGT | Regulation |
|  | YE1436 R | TTGCGGATTCAACCATTCCA |  |
| *fleC*, Flagellin FleC | FleC F | GAGCCAGAACAGAAGTACCG | Motility |
|  | FleC R | CACCATCACCAACCTGAACA |  |
| *fliS*, Putative cytoplasmic chaperone | FliS F | GGTGAGCTGGCAGAGAATTT | Motility |
|  | FliS R | TGCCTGTTCGTCATTATGCA |  |
| YE2848, Putative chemotaxis methyl-accepting transducer | YE2848 F | TCCTGAGACTGCAACATTCG | Motility |
|  | YE2848 R | ATTCGCAAAGACGGCAGTAT |  |

**Reference**

Bresolin G, Neuhaus K, Scherer S, Fuchs TM. 2006. Transcriptional analysis of long-term adaptation of Yersinia enterocolitica to low-temperature growth. Journal of bacteriology. Apr;188:2945-2958.
