# Supplementary material for: Isolate Specific Cold Response of Yersinia enterocolitica in Transcriptional, Proteomic, and Membrane Physiological Changes
Source: Front Microbiol. 2020 Jan 23;10:3037. doi: 10.3389/fmicb.2019.03037 (PMC6990146; doi:10.3389/fmicb.2019.03037)
Supplement: Supplementary file 3 [file Table_3.docx]

**S3. Membrane related genes detected in *Y. enterocolitica* strains**

| **Target gene** | **Protein** | **Function** | **Pimer (5’ – 3’ sequence)** | **Reference** |
| --- | --- | --- | --- | --- |
| *ompF* | Outer membrane porin protein F | Stress response depended | F: GTTTTGTTGCCTTTGTCGCC | [[1](#_ENREF_1), [2](#_ENREF_2)] |
|  |  |  | R: CGTTTCGGCTTCAAAGGTGA |  |
| *ompA* | Outer membrane protein A | Membrane protein | F: TATTCTCCTTCAGCCACGGG | [[3](#_ENREF_3), [4](#_ENREF_4)] |
|  |  |  | R: TAGACCAATGCAGCGACAGA |  |
| *ompC* | Outer membrane porin protein C | Stress response depended | F: TACTTACGGTGCGGACAACT | [[1](#_ENREF_1), [2](#_ENREF_2)] |
|  |  |  | R: TACCGTAGCCGTCACCATTT |  |
| *ompR* | Transcriptional regulator | Required for the transcriptional expression of *ompF* and *ompC* | F: ATGGAACGCTCTATCGACGT | [[5](#_ENREF_5), [6](#_ENREF_6)] |
|  |  |  | R: CGTCCGGCACAAATACGTAG |  |
| *fadL* | Outer membrane transporter | Long-chain fatty acid transporter | F: GCGGTGGTAATTGGCTACTG | [[7](#_ENREF_7), [8](#_ENREF_8)] |
|  |  |  | R: AAGCTGGTGGTGGACGATTA |  |
| *yaeT* | Outer membrane protein assembly factor | Lipoprotein | F: ACCCGCTAGTTTCTGCTTCT | [[9](#_ENREF_9), [10](#_ENREF_10)] |
|  |  |  | R: CAGTGAAGGCTGTCGTTACG |  |
| *yfgL* | Outer membrane protein assembly factor | Lipoprotein | F: GCCACTGCCAAAAGTTGAGA | [[9](#_ENREF_9), [10](#_ENREF_10)] |
|  |  |  | R: ACGCCACCAGATAACATTGC |  |
| *dapX* | Outer membrane protein assembly factor | Lipoprotein | F: GATGTCCGCTCTGTCAACAC | [[9](#_ENREF_9), [10](#_ENREF_10)] |
|  |  |  | R: CCAGTCAGTTGTCAGGGTCT |  |
| *yfiO* | Outer membrane protein assembly factor | Lipoprotein | F: GTCAGTTGCGTATTGGCTGT | [[9](#_ENREF_9), [10](#_ENREF_10)] |
|  |  |  | R: TGCGTGGTTTGACTGACATG |  |
| *pldA* | Outer membrane phospholipase A | Outer membrane phospholipase A | F: GCTGGGATCGCGTTTATACC | [[11](#_ENREF_11), [12](#_ENREF_12)] |
|  |  |  | R: AACACACTTTCACCCAAGGC |  |
| *tolC* | Outer membrane channel protein | Outer membrane channel protein | F: ATGAGCCAGGCAGAGAACTT | [[13](#_ENREF_13)] |
|  |  |  | R: TGTAACCCGCACCTAAACCT |  |
| *lpxP* | Lipid A biosynthesis palmitoleoyl transferase | Fluidity generated | F: TGCAGGCGCTGAAGAAAGG | [[14](#_ENREF_14), [15](#_ENREF_15)] |
|  |  |  | R: TAAAGGTGCCGCTGGTGGT |  |
| *lpxM* | Lipid A biosynthesis (KDO)2-(lauroyl)-lipid IVA acyltransferase | Fluidity generated | F: GGCATATGTCCCACCGAAAC | [[14](#_ENREF_14), [16](#_ENREF_16)] |
|  |  |  | R: CACTTTCTTTGGGTCGCGAA |  |
| *lpxL* | Lipid A biosynthesis lauroyl acyltransferase | Fluidity generated | F: TCACTACTTCACCCTCGTTAT | [[14](#_ENREF_14), [16](#_ENREF_16)] |
|  |  |  | R: TCTTTAGCAGCAGTTCATCTC |  |
| *des* | Fatty acid desaturase | Fluidity related | F: TAGGCACAGTGCTAGGAAG | [[17](#_ENREF_17), [18](#_ENREF_18)] |
|  |  |  | R: ATGGTGTTGAATATGGGAG |  |

1. Begic, S. and E.A. Worobec, *Regulation of Serratia marcescens ompF and ompC porin genes in response to osmotic stress, salicylate, temperature and pH.* Microbiology, 2006. **152**(Pt 2): p. 485-91.

2. Luokkamäki, M. and E.T. Palva, *Cold-sensitive ompB mutants affecting expression of ompC in Escherichia coli K12.* FEMS Microbiology Letters, 1987. **40**(1): p. 21-25.

3. Wang, Y., *The Function of OmpA in Escherichia coli.* Biochemical and Biophysical Research Communications, 2002. **292**(2): p. 396-401.

4. Taura, T., T. Yoshihisa, and K. Ito, *Protein translocation functions of Escherichia coli SecY: in vitro characterization of cold-sensitive secY mutants.* Biochimie, 1997. **79**(8): p. 517-21.

5. Bang, I.S., et al., *OmpR regulates the stationary-phase acid tolerance response of Salmonella enterica serovar typhimurium.* Journal of bacteriology, 2000. **182**(8): p. 2245-2252.

6. Jaworska, K., et al., *OmpR-Mediated Transcriptional Regulation and Function of Two Heme Receptor Proteins of Yersinia enterocolitica Bio-Serotype 2/O:9.* Frontiers in Cellular and Infection Microbiology, 2018. **8**(333).

7. Lepore, B.W., et al., *Ligand-gated diffusion across the bacterial outer membrane.* Proceedings of the National Academy of Sciences, 2011. **108**(25): p. 10121-10126.

8. DiRusso, C.C. and P.N. Black, *Bacterial long chain fatty acid transport: gateway to a fatty acid-responsive signaling system.* Journal of Biological Chemistry, 2004. **279**(48): p. 49563-49566.

9. Sklar, J.G., et al., *Lipoprotein SmpA is a component of the YaeT complex that assembles outer membrane proteins in Escherichia coli.* Proceedings Of the National Academy Of Sciences Of the United States Of America, 2007. **104**(15): p. 6400-6405.

10. Rigel, N.W. and T.J. Silhavy, *Making a beta-barrel: assembly of outer membrane proteins in Gram-negative bacteria.* Curr Opin Microbiol, 2012. **15**(2): p. 189-93.

11. Belosludtsev, K., et al., *Interaction of Phospholipase A of the E. coli Outer Membrane with the Inhibitors of Eucaryotic Phospholipases A(2) and Their Effect on the Ca2+-Induced Permeabilization of the Bacterial Membrane.* The Journal of membrane biology, 2014. **247**.

12. Dekker, N., *Outer-membrane phospholipase A: known structure, unknown biological function.* Molecular Microbiology, 2000. **35**(4): p. 711-717.

13. Werner, J., A.M. Augustus, and R. Misra, *Assembly of TolC, a Structurally Unique and Multifunctional Outer Membrane Protein of <em>Escherichia coli</em> K-12.* Journal of Bacteriology, 2003. **185**(22): p. 6540-6547.

14. Carty, S.M., K.R. Sreekumar, and C.R. Raetz, *Effect of Cold Shock on Lipid A Biosynthesis inEscherichia coli INDUCTION AT 12° C OF AN ACYLTRANSFERASE SPECIFIC FOR PALMITOLEOYL-ACYL CARRIER PROTEIN.* Journal of Biological Chemistry, 1999. **274**(14): p. 9677-9685.

15. Vorachek-Warren, M.K., et al., *An Escherichia coli mutant lacking the cold shock-induced palmitoleoyltransferase of lipid A biosynthesis: absence of unsaturated acyl chains and antibiotic hypersensitivity at 12 degrees C.* J Biol Chem, 2002. **277**(16): p. 14186-93.

16. van der Ley, P., et al., *Modification of lipid A biosynthesis in Neisseria meningitidis lpxL mutants: influence on lipopolysaccharide structure, toxicity, and adjuvant activity.* Infection and immunity, 2001. **69**(10): p. 5981-5990.

17. Aguilar, P.S., et al., *Molecular basis of thermosensing: a two-component signal transduction thermometer in Bacillus subtilis.* The EMBO journal, 2001. **20**(7): p. 1681-1691.

18. Albanesi, D., M.C. Mansilla, and D. de Mendoza, *The Membrane Fluidity Sensor DesK of Bacillus subtilis Controls the Signal Decay of Its Cognate Response Regulator.* Journal of Bacteriology, 2004. **186**(9): p. 2655-2663.
