# Supplementary material for: Isolate Specific Cold Response of Yersinia enterocolitica in Transcriptional, Proteomic, and Membrane Physiological Changes
Source: Front Microbiol. 2020 Jan 23;10:3037. doi: 10.3389/fmicb.2019.03037 (PMC6990146; doi:10.3389/fmicb.2019.03037)
Supplement: Supplementary file 4 [file Table_4.DOCX]

**S4. List of the differentially expressed proteins involved in cold responses.** Protein entry is the accession number and proteins names are as listed in the Uniprot Knowledgebase database. Regulation: only those proteins which are signiﬁcantly regulated after ﬁltering 1% false discovery rate (FDR). (+) indicates upregulated and (−) indicates downregulated.

| **Protein entry** | **Gene names** | **Protein names** | **Regulation** | | | | **Functional annotation in**  **KEGG pathway** | **Sources and references** | | |
| --- | --- | --- | --- | --- | --- | --- | --- | --- | --- | --- |
|  |  |  | **44B-T1** | **44B-T2** | **II7D-T1** | **II7D-T2** |  |  |  |  |
| **Energy production and conversion, and carbohydrate transport and metabolism** | | | | | | | | | | |
| A0A0E8M4Z5 | *tpiA* | Triosephosphate isomerase |  |  |  | (+) | Glycolysis / Gluconeogenesis | *Moraxella catarrhalis* | [[1](#_ENREF_1)] | |
| A0A0E1NJ48 | *pykF* | Pyruvate kinase |  |  |  | (+) | Glycolysis / Gluconeogenesis | *Escherichia. coli* | [[2](#_ENREF_2)] | |
| A0A0E1NLE9 | *glcA* | PTS system glucose-specific transporter subunit IIBC |  |  |  | (+) | Glycolysis / Gluconeogenesis | *E. coli* | [[3](#_ENREF_3)] | |
| A0A0E8HAK8 | *adhE* | Aldehyde dehydrogenase |  |  |  | (+) | Glycolysis / Gluconeogenesis | *Lactococcus piscium*, *E. coli* | [[4](#_ENREF_4), [5](#_ENREF_5)], | |
| A0A0U1H9E9 | *yeaD* | Putative glucose-6-phosphate 1-epimerase |  |  |  | (+) | Glycolysis / Gluconeogenesis | This study |  | |
| A0A0U1H9E5 | *acnB* | Aconitate hydratase B |  |  |  | (+) | Propanoate metabolism | *M. catarrhalis* | [[1](#_ENREF_1)] | |
| A0A0U1H9X1 | *gldA* | Glycerol dehydrogenase |  |  | (+) | (+) | Propanoate metabolism | *Halobacterium* | [[6](#_ENREF_6)] | |
| A0A0F6WY02 | *yqhD* | Putative iron-containing alcohol dehydrogenase |  |  |  | (+) | Propanoate metabolism | *E. coli* | [[7](#_ENREF_7)] | |
| A0A0E1NCT9 | *sdhB* | Succinate dehydrogenase iron-sulfur subunit |  |  |  | (+) | Pyruvate metabolism | *Pseudoalteromonas haloplanktis* | [[8](#_ENREF_8)] | |
| A0A0E8HWV3 | *pflB* | Formate acetyltransferase 1 |  |  |  | (+) | Pyruvate metabolism | This study |  | |
| A0A0T9SI31 | *ppc* | Phosphoenolpyruvate carboxylase |  |  |  | (+) | Pyruvate metabolism | This study |  | |
| A0A0E8LXM1 | *cydA* | Cytochrome D ubiquinol oxidase subunit I |  |  |  | (+) | Oxidative phosphorylation | *E. coli* | [[5](#_ENREF_5)] | |
| A0A0E1NAF3 | *atpF* | F-ATPase subunit b |  |  |  | (+) | Oxidative phosphorylation | *Bacillus subtilis* | [[9](#_ENREF_9)] | |
| A0A0E1NGF7 | *eda* | keto-deoxy-phosphogluconate aldolase |  |  |  | (+) | Pentose phosphate pathway | This study |  | |
| A0A0F6SVL1 | *gntK* | Gluconokinase |  | (+) |  |  | Pentose phosphate pathway | This study |  | |
| A0A0E8HH58 | *mtlA* | PTS system mannitol-specific transporter subunit IICBA |  |  |  | (+) | Fructose and mannose metabolism | This study |  | |
| A0A0U1H7P3 | *fruA* | PTS system fructose-specific transporter subunit IIBC |  |  |  | (+) | Fructose and mannose metabolism | This study |  | |
| A0A0E8K0D6 | *galA* | Galactokinase |  |  |  | (+) | Amino sugar and nucleotide sugar metabolism | This study |  | |
| A0A0U1HJ22 | *gabD* | Succinate-semialdehyde dehydrogenase |  |  | (+) | (+) | Butanoate metabolism | This study |  | |
| A0A0U1HEM1 | *gor* | Glutathione reductase |  |  |  | (+) | Glutathione metabolism | This study |  | |
| A0A0N7FFD3 | *glpQ* | Glycerophosphodiester phosphodiesterase | (+) |  |  | (-) | Glycerophospholipid metabolism | *P. haloplanktis* | [[10](#_ENREF_10)] | |
| A0A0F6SVL0 | *iolE* | 2-keto-myo-inositol dehydratase | (+) |  |  |  | Inositol phosphate metabolism | *Lactobacillus* | [[11](#_ENREF_11)] | |
| **Transcription, translation, ribosomal structure and biogenesis, and posttranslational modification, protein turnover, chaperones** | | | | | | | | | | |
| A0A0E1NLF9 | *nusA* | Transcription termination/antitermination protein NusA |  |  |  | (+) | Ribosome Biogenesis | *Shewanella oneidensis*，*E. coli* | | [[12](#_ENREF_12), [13](#_ENREF_13)]， |
| A0A0E1NFQ2 | *raiA* | ribosome-associated inhibitor A |  | (+) |  |  | Ribosome Biogenesis | *E. coli* | | [[14](#_ENREF_14)] |
| A0A0N9KYC5 | *rsmB* | Ribosomal RNA small subunit methyltransferase B |  |  |  | (+) | Ribosome Biogenesis | *E. coli* | | [[15](#_ENREF_15)] |
| A0A0E8J898 | *rsmI* | Ribosomal RNA small subunit methyltransferase I |  |  |  | (+) | Ribosome Biogenesis | This study | |  |
| A0A0E8GHX6 | *rsmC* | Ribosomal RNA small subunit methyltransferase C |  |  |  | (+) | Ribosome Biogenesis | This study | |  |
| A0A0F6X0D9 | *trmG* | Putative RNA methyltransferase |  |  |  | (+) | Ribosome Biogenesis | This study | |  |
| A0A0E1NG62 | *rsfS* | Ribosomal silencing factor RsfS |  | (+) |  | (+) | Ribosome Biogenesis | This study | |  |
| A0A0E8GRF3 | *hflX* | GTPase HflX |  |  |  | (+) | Ribosome Biogenesis | This study | |  |
| A0A0E8JF17 | *engD* | Ribosome-binding ATPase YchF |  |  |  | (+) | Ribosome Biogenesis | This study | |  |
| A0A0E8KN19 | *rluF* | Pseudouridine synthase |  |  |  | (+) | Ribosome Biogenesis | This study | |  |
| A0A0E1NL30 | *rsuA* | Pseudouridine synthase |  |  |  | (+) | Ribosome Biogenesis | This study | |  |
| A0A0E1NBS8 | *lexA* | LexA repressor |  |  | (+) | (+) | Peptidases and Inhibitor | *S. piezotolerans* | | [[16](#_ENREF_16)] |
| A0A0F6WY61 | *yggG* | Putative lipoprotein |  | (+) |  |  | Peptidases and Inhibitor | *E. coli* | | [[5](#_ENREF_5)] |
| A0A0E8GVJ3 | *hflK* | Protein HflK |  |  |  | (+) | Peptidases and Inhibitor | *E. coli* | | [[15](#_ENREF_15)] |
| A0A0E1NFT2 | *hflC* | Protein HflC |  |  |  | (+) | Peptidases and Inhibitor | This study | |  |
| A0A0E8HBU0 | *sppA* | Signal peptide peptidase |  |  |  | (+) | Peptidases and Inhibitor | This study | |  |
| A0A0E8GKB5 | *nhaR* | Transcriptional activator |  |  | (+) | (+) | Transcription Factors | *E. coli* | | [[3](#_ENREF_3)] |
| A0A0E8M8P5 | *oxyR* | DNA-binding transcriptional regulator |  |  |  | (+) | Transcription Factors | *Vibrio vulnificus* | | [[17](#_ENREF_17)] |
| A0A0E8MDX9 | *fadR* | Fatty acid metabolism regulator protein |  |  |  | (+) | Transcription Factors | *M. catarrhalis* | | [[1](#_ENREF_1)] |
| A0A0E1NDV1 | *cysB* | Transcriptional regulator |  |  |  | (+) | Transcription Factors | This study | |  |
| A0A0E8M7I2 | *rfaH* | Transcription antitermination protein |  |  |  | (+) | Transcription Factors | This study | |  |
| A0A0E8LEJ7 | *trpS* | Tryptophanyl-tRNA synthetase |  | (-) |  | (+) | Aminoacyl-tRNA biosynthesis | This study | |  |
| A0A0U1HAK8 | *lysS1* | Lysyl-tRNA synthetase |  |  |  | (+) | Aminoacyl-tRNA biosynthesis | This study | |  |
| A0A0U1HAQ9 | *lysS2* | Lysyl-tRNA synthetase |  |  |  | (+) | Aminoacyl-tRNA biosynthesis | This study | |  |
| A0A0E1NFL7 | *rho* | Transcription termination factor Rho |  |  |  | (+) | RNA degradation | *Caulobacter crescentus* | | [[18](#_ENREF_18)] |
| A0A0N9KFU9 | *rnr* | Ribonuclease R |  |  |  | (+) | RNA degradation | *E. coli* | | [[19](#_ENREF_19)] |
| A0A0E1NGJ5 | *pnp* | Polyribonucleotide nucleotidyltransferase |  |  |  | (+) | RNA degradation | *E. coli* | | [[20](#_ENREF_20)] |
| A0A0E1NF11 | *rpoD* | RNA polymerase sigma factor |  |  |  | (+) | Transcription Machinery | *E. coli* | | [[21](#_ENREF_21)] |
| A0A0F6WXY2 | *sspA* | Stringent starvation protein A |  |  |  | (+) | Transcription Machinery | This study | |  |
| A0A0E1N9D4 | *clpA* | ATP-dependent Clp protease ATP-binding subunit |  |  |  | (+) | Chaperones and Folding Catalysts | *E. coli* | | [[22](#_ENREF_22)] |
| A0A0E1N9K3 | *dnaJ* | Chaperone protein DnaJ |  |  | (+) |  | Chaperones and Folding Catalysts | *E. coli* | | [[23](#_ENREF_23)] |
| A0A0E8L6H5 | *mnmA* | tRNA-specific 2-thiouridylase |  |  |  | (+) | Sulfur relay system | *L. piscium, E. coli* | | [[4](#_ENREF_4), [5](#_ENREF_5)] |
| A0A0U1H910 | *rnd* | Ribonuclease D |  | (+) |  |  | Sulfur relay system | This study | |  |
| A0A0E8GPP3 | *infB* | Translation initiation factor IF-2 |  |  | (+) | (+) | Ribosome | *E. coli*, *B. subtilis* | | [[24](#_ENREF_24), [25](#_ENREF_25)] |
| A0A0E1NCR6 | *speG* | Spermidine acetyltransferase |  |  | (+) |  | Arginine and proline metabolism | E. coli | | [[18](#_ENREF_18)] |
| **Amino acid transport and metabolism** | | | | | | | | | | |
| A0A0E8H7I5 | *trpB* | Tryptophan synthase beta chain |  |  |  | (+) | Glycine, serine and threonine metabolism | *E. coli*, *B. subtilis* | | [[3](#_ENREF_3), [26](#_ENREF_26)] |
| A0A0U1HAY8 | *gcvP* | Glycine dehydrogenase |  |  | (+) |  | Glycine, serine and threonine metabolism | *E. coli* | | [[27](#_ENREF_27)] |
| A0A0T9SBT5 | *ilvA* | L-threonine dehydratase |  | (+) | (+) |  | Glycine, serine and threonine metabolism | *Staphylococcus aureus* | | [[28](#_ENREF_28)] |
| A0A0U1HHS7 | *tdh* | L-threonine 3-dehydrogenase |  |  |  | (+) | Glycine, serine and threonine metabolism | *Vibrio parahaemolyticus* | | [[29](#_ENREF_29)] |
| A0A0T9SUV2 | *sdaA* | L-serine dehydratase |  |  |  | (+) | Glycine, serine and threonine metabolism | This study | |  |
| A0A0U1H959 | *dsdA* | D-serine dehydratase |  |  |  | (+) | Glycine serine and threonine metabolism | This study | |  |
| A0A0U1HJX6 | *tdcB* | Pyridoxal-phosphate dependent protein |  |  |  | (+) | Glycine serine and threonine metabolism | This study | |  |
| A0A0F6SXG4 | *leuA* | 2-isopropylmalate synthase |  | (+) |  |  | Valine, leucine and isoleucine biosynthesis | *E. coli, B. subtilis, Thermoanaerobacter tengcongensis* | | [[27](#_ENREF_27), [30](#_ENREF_30), [31](#_ENREF_31)] |
| A0A0F6ZK76 | *leuB* | 3-isopropylmalate dehydrogenase |  | (+) |  |  | Valine, leucine and isoleucine biosynthesis | *E. coli, B. subtilis, T. tengcongensis* | | [[27](#_ENREF_27), [30](#_ENREF_30), [31](#_ENREF_31)] |
| A0A0F6ZMQ3 | *leuC* | 3-isopropylmalate dehydratase large subunit |  | (+) |  |  | Valine, leucine and isoleucine biosynthesis | *B. subtilis, T. tengcongensis* | | [[30](#_ENREF_30), [31](#_ENREF_31)] |
| A0A0T9RBA5 | *leuD* | 3-isopropylmalate dehydratase small subunit |  | (+) |  |  | Valine, leucine and isoleucine biosynthesis | *B. subtilis, T. tengcongensis* | | [[30](#_ENREF_30), [31](#_ENREF_31)] |
| A0A0F6ZG28 | *budB* | Acetolactate synthase |  |  |  | (+) | Valine, leucine and isoleucine biosynthesis | *B. subtilis* | | [[32](#_ENREF_32)] |
| A0A0U1HG86 | *aat* | Aminotransferase |  |  |  | (+) | Valine, leucine and isoleucine biosynthesis | This study | |  |
| A0A0F6ZFK3 | *argD* | Acetylornithine/succinyldiaminopimelate aminotransferase |  |  |  | (+) | Arginine biosynthesis | *E. coli* | | [[5](#_ENREF_5)] |
| A0A0E8GMP3 | *arcA* | Arginine deiminase |  |  |  | (+) | Arginine biosynthesis | *Vibrio parahaemolyticus* | | [[29](#_ENREF_29)] |
| A0A0U1HHW8 | *argE* | Acetylornithine deacetylase |  |  |  | (+) | Arginine biosynthesis | This study | |  |
| A0A0U1HAL7 | *proC* | Pyrroline-5-carboxylate reductase |  |  |  | (+) | Arginine and proline metabolism | This study | |  |
| A0A0F6WZZ5 | *hisA* | Phosphoribosylformimino-5-aminoimidazole carboxamide ribotide isomerase |  |  |  | (+) | Histidine metabolism | *E. coli* | | [[21](#_ENREF_21)] |
| A0A0E8JVR4 | *hisB* | Histidine biosynthesis bifunctional protein HisB |  |  |  | (+) | Histidine metabolism | *E. coli* | | [[21](#_ENREF_21)] |
| A0A0U1HDG1 | *hisI* | Phosphoribosyl-AMP cyclohydrolase |  | (+) |  |  | Histidine metabolism | *E. coli* | | [[21](#_ENREF_21)] |
| A0A0U1HDR3 | *hisH* | Imidazole glycerol phosphate synthase subunit |  |  |  | (+) | Histidine metabolism | *E. coli* | | [[21](#_ENREF_21)] |
| A0A0U1HLH3 | *pheA* | prephenate dehydratase |  | (+) |  |  | Phenylalanine, tyrosine and tryptophan biosynthesis | *E. coli* | | [[33](#_ENREF_33)] |
| A0A0T9R5Q0 | *aroF* | Phospho-2-dehydro-3-deoxyheptonate aldolase |  |  |  | (+) | Phenylalanine, tyrosine and tryptophan biosynthesis | *E. coli* | | [[22](#_ENREF_22)] |
| A0A0E8LHW0 | *aroB* | 3-dehydroquinate synthase |  |  |  | (+) | Phenylalanine, tyrosine and tryptophan biosynthesis | This study | |  |
| A0A0U1HGD1 | *aroC* | Chorismate synthase |  |  |  | (+) | Phenylalanine, tyrosine and tryptophan biosynthesis | This study | |  |
| A0A0F6X0U4 | *speF1* | Ornithine decarboxylase |  |  | (+) | (+) | Glutathione metabolism | This study | |  |
| A0A0F6ZFY3 | *speF2* | Ornithine decarboxylase |  |  | (+) | (+) | Glutathione metabolism | This study | |  |
| A0A0U1HB41 | *speC* | Ornithine decarboxylase |  |  |  | (+) | Glutathione metabolism | This study | |  |
| A0A0E8GLD1 | *pepA* | Probable cytosol aminopeptidase |  |  |  | (+) | Glutathione metabolism | This study | |  |
| A0A0U1HA57 | *carA* | Carbamoyl-phosphate synthase small chain |  |  |  | (+) | Alanine, aspartate and glutamate metabolism | *B. subtilis* | | [[25](#_ENREF_25)] |
| A0A0E8M0L1 | *asnB* | Asparagine synthetase B |  |  |  | (+) | Alanine, aspartate and glutamate metabolism | *E. coli* | | [[2](#_ENREF_2), [5](#_ENREF_5)] |
| A0A0E8HPB5 | *asnA* | Aspartate-ammonia ligase |  |  | (+) |  | Alanine, aspartate and glutamate metabolism | *E. coli* | | [[2](#_ENREF_2)] |
| **Cell wall/membrane/envelope biogenesis and cell motility** | | | | | | | | | | |
| A0A0U1HA39 | *lpxB* | Lipid-A-disaccharide synthase |  |  |  | (+) | Lipopolysaccharide biosynthesis | *M. catarrhalis* | | [[1](#_ENREF_1)] |
| A0A0U1HAB3 | *lpxA* | UDP-N-acetylglucosamine acyltransferase |  |  |  | (+) | Lipopolysaccharide biosynthesis | *M. catarrhalis* | | [[6](#_ENREF_6)] |
| A0A0E8JAG1 | *lpxP* | Lipid A biosynthesis palmitoleoyltransferase |  |  |  | (+) | Lipopolysaccharide biosynthesis | *E. coli* | | [[34](#_ENREF_34)] [[35](#_ENREF_35)] |
| A0A0F6SWK1 | *lpxM* | Lipid A biosynthesis myristoyltransferase |  |  |  | (+) | Lipopolysaccharide biosynthesis | *E. coli*, *Neisseria meningitidis* | | [[34](#_ENREF_34), [36](#_ENREF_36)] |
| A0A0F6X147 | *rfaC* | ADP-heptose--LPS heptosyltransferase |  |  |  | (+) | Lipopolysaccharide biosynthesis | This study | |  |
| A0A0N9K223 | *rfaQ* | Putative lipopolysaccharide core biosynthesis protein |  |  |  | (+) | Lipopolysaccharide biosynthesis | This study | |  |
| A0A0F6ZMP8 | *ddl* | D-alanine-D-alanine ligase |  |  | (+) |  | Peptidoglycan biosynthesis | *Yersinia pestis* | | [[37](#_ENREF_37)] |
| A0A0E1NJT4 | *dacB* | D-alanyl-D-alanine carboxypeptidase |  |  |  | (+) | Peptidoglycan biosynthesis | This study | |  |
| A0A0U1H9K3 | *ftsI* | Penicillin-binding protein 3 |  |  |  | (+) | Peptidoglycan biosynthesis | This study | |  |
| A0A0U1H9N3 | *murC* | UDP-N-acetylmuramoyl-L-alanine synthetase |  |  |  | (+) | Peptidoglycan biosynthesis | This study | |  |
| A0A0U1H9I0 | *murE* | UDP-N-acetylmuramyl-tripeptide synthetase |  |  |  | (+) | Peptidoglycan biosynthesis | This study | |  |
| A0A0U1HJ04 | *mrcA* | Peptidoglycan synthetase |  |  |  | (+) | Peptidoglycan biosynthesis | This study | |  |
| A0A0E8HQK5 | *glmS* | Hexosephosphate aminotransferase |  |  |  | (+) | Amino sugar and nucleotide sugar metabolism | This study | |  |
| A0A0E1NEL7 | *galD* | UDP-galactose-4-epimerase |  |  |  | (+) | Amino sugar and nucleotide sugar metabolism | This study | |  |
| A0A0E1NEU3 | *wecC* | UDP-N-acetyl-D-mannosamine dehydrogenase |  | (-) |  | (+) | Amino sugar and nucleotide sugar metabolism | This study | |  |
| A0A0U1HEJ0 | *arnA* | UDP-L-Ara4N formyltransferase |  |  |  | (+) | Amino sugar and nucleotide sugar metabolism | This study | |  |
| A0A0U1HEG5 | *arnB* | UDP-4-amino-4-deoxy-L-arabinose--oxoglutarate aminotransferase |  |  |  | (+) | Amino sugar and nucleotide sugar metabolism | This study | |  |
| A0A0E8KE62 | *dapX* | Outer membrane protein assembly factor |  | (+) |  |  | Transporters | This study | |  |
| A0A0E8M1X9 | *excC* | Peptidoglycan-associated outer membrane lipoprotein |  | (+) |  |  | Transporters | *Y. pestis* | | [[38](#_ENREF_38)] |
| A0A0E1NKL7 | *yidC* | Membrane protein insertase |  |  |  | (+) | Quorum sensing | *E. coli* | | [[39](#_ENREF_39)] |
| A0A0U1HIG4 | *pldA* | Phospholipase A1 |  | (+) |  |  | Glycerophospholipid metabolism | *E. coli* | | [[40](#_ENREF_40), [41](#_ENREF_41)] |
| A0A0U1HGP8 | *trg* | Putative methyl-accepting chemotaxis protein |  |  |  | (+) | Bacterial chemotaxis | *S. oneidensis* | | [[12](#_ENREF_12)] |
| A0A0E1NK07 | *cheB* | Protein-glutamate methylesterase |  |  |  | (+) | Bacterial chemotaxis | *T. tengcongensis* | | [[31](#_ENREF_31)] |
| A0A0E8LV00 | *cheD* | Methyl-accepting chemotaxis protein |  |  |  | (+) | Bacterial chemotaxis | This study | |  |
| Q93P01 | *cheZ* | CheZ |  |  |  | (+) | Bacterial chemotaxis | This study | |  |

1. Spaniol, V., S. Wyder, and C. Aebi, *RNA-Seq-based analysis of the physiologic cold shock-induced changes in Moraxella catarrhalis gene expression.* PLoS One, 2013. **8**(7): p. e68298.

2. Gadgil, M., V. Kapur, and W.S. Hu, *Transcriptional response of Escherichia coli to temperature shift.* Biotechnol Prog, 2005. **21**(3): p. 689-99.

3. White-Ziegler, C.A., et al., *Low temperature (23 degrees C) increases expression of biofilm-, cold-shock- and RpoS-dependent genes in Escherichia coli K-12.* Microbiology, 2008. **154**(Pt 1): p. 148-66.

4. Garnier, M., et al., *Adaptation to cold and proteomic responses of the psychrotrophic biopreservative Lactococcus piscium strain CNCM I-4031.* Appl Environ Microbiol, 2010. **76**(24): p. 8011-8.

5. Phadtare, S. and M. Inouye, *Genome-wide transcriptional analysis of the cold shock response in wild-type and cold-sensitive, quadruple-csp-deletion strains of Escherichia coli.* Journal of bacteriology, 2004. **186**(20): p. 7007-7014.

6. Coker, J.A., et al., *Transcriptional profiling of the model Archaeon Halobacterium sp. NRC-1: responses to changes in salinity and temperature.* Saline systems, 2007. **3**: p. 6-6.

7. Perez, J.M., et al., *Escherichia coli YqhD exhibits aldehyde reductase activity and protects from the harmful effect of lipid peroxidation-derived aldehydes.* J Biol Chem, 2008. **283**(12): p. 7346-53.

8. Piette, F., et al., *Life in the cold: a proteomic study of cold-repressed proteins in the antarctic bacterium pseudoalteromonas haloplanktis TAC125.* Appl Environ Microbiol, 2011. **77**(11): p. 3881-3.

9. Beckering, C.L., et al., *Genomewide transcriptional analysis of the cold shock response in Bacillus subtilis.* J Bacteriol, 2002. **184**(22): p. 6395-402.

10. Piette, F., et al., *Life in the cold: a proteomic study of cold-repressed proteins in the Antarctic bacterium Pseudoalteromonas haloplanktis TAC125.* Appl. Environ. Microbiol., 2011. **77**(11): p. 3881-3883.

11. Monedero, V., et al., *The phosphotransferase system of Lactobacillus casei: regulation of carbon metabolism and connection to cold shock response.* J Mol Microbiol Biotechnol, 2007. **12**(1-2): p. 20-32.

12. Gao, H., et al., *Global transcriptome analysis of the cold shock response of Shewanella oneidensis MR-1 and mutational analysis of its classical cold shock proteins.* J Bacteriol, 2006. **188**(12): p. 4560-9.

13. Li, K., et al., *Escherichia coli transcription termination factor NusA: heat-induced oligomerization and chaperone activity.* Sci Rep, 2013. **3**: p. 2347.

14. Di Pietro, F., et al., *Role of the ribosome-associated protein PY in the cold-shock response of Escherichia coli.* Microbiologyopen, 2013. **2**(2): p. 293-307.

15. Burakovsky, D.E., et al., *Impact of methylations of m2G966/m5C967 in 16S rRNA on bacterial fitness and translation initiation.* Nucleic Acids Res, 2012. **40**(16): p. 7885-95.

16. Jian, H., et al., *The regulatory function of LexA is temperature-dependent in the deep-sea bacterium Shewanella piezotolerans WP3.* Frontiers in microbiology, 2015. **6**: p. 627-627.

17. Limthammahisorn, S., Y.J. Brady, and C.R. Arias, *Gene expression of cold shock and other stress-related genes in Vibrio vulnificus grown in pure culture under shellstock temperature control conditions.* J Food Prot, 2008. **71**(1): p. 157-64.

18. Aguirre, A.A., et al., *Association of the Cold Shock DEAD-Box RNA Helicase RhlE to the RNA Degradosome in Caulobacter crescentus.* J Bacteriol, 2017. **199**(13).

19. Arraiano, C.M. and L.E. Maquat, *Post-transcriptional control of gene expression: effectors of mRNA decay.* Molecular Microbiology, 2003. **49**(1): p. 267-276.

20. Yamanaka, K. and M. Inouye, *Selective mRNA degradation by polynucleotide phosphorylase in cold shock adaptation in Escherichia coli.* Journal of bacteriology, 2001. **183**(9): p. 2808-2816.

21. King, T., et al., *Physiological Response of Escherichia coli O157:H7 Sakai to Dynamic Changes in Temperature and Water Activity as Experienced during Carcass Chilling.* Molecular & cellular proteomics : MCP, 2016. **15**(11): p. 3331-3347.

22. Lee, S.J., et al., *Family of the major cold-shock protein, CspA (CS7.4), of Escherichia coli, whose members show a high sequence similarity with the eukaryotic Y-box binding proteins.* Molecular Microbiology, 1994. **11**(5): p. 833-839.

23. Schroder, H., et al., *DnaK, DnaJ and GrpE form a cellular chaperone machinery capable of repairing heat-induced protein damage.* EMBO J, 1993. **12**(11): p. 4137-44.

24. Brandi, A., et al., *Transcriptional and post-transcriptional events trigger de novo infB expression in cold stressed Escherichia coli.* Nucleic Acids Res, 2019. **47**(9): p. 4638-4651.

25. Beckering, C.L., et al., *Genomewide transcriptional analysis of the cold shock response in Bacillus subtilis.* Journal of bacteriology, 2002. **184**(22): p. 6395-6402.

26. Budde, I., et al., *Adaptation of Bacillus subtilis to growth at low temperature: a combined transcriptomic and proteomic appraisal.* Microbiology, 2006. **152**(3): p. 831-853.

27. Phadtare, S., *Escherichia coli cold-shock gene profiles in response to over-expression/deletion of CsdA, RNase R and PNPase and relevance to low-temperature RNA metabolism.* Genes to cells : devoted to molecular & cellular mechanisms, 2012. **17**(10): p. 850-874.

28. Anderson, K.L., et al., *Characterization of the Staphylococcus aureus heat shock, cold shock, stringent, and SOS responses and their effects on log-phase mRNA turnover.* Journal of bacteriology, 2006. **188**(19): p. 6739-6756.

29. Zhu, C., et al., *Genomic and transcriptomic analyses reveal distinct biological functions for cold shock proteins (VpaCspA and VpaCspD) in Vibrio parahaemolyticus CHN25 during low-temperature survival.* BMC genomics, 2017. **18**(1): p. 436-436.

30. Kaan, T., et al., *Genome-wide transcriptional profiling of the Bacillus subtilis cold-shock response.* Microbiology, 2002. **148**(11): p. 3441-3455.

31. Liu, B., Y. Zhang, and W. Zhang, *RNA-Seq-based analysis of cold shock response in Thermoanaerobacter tengcongensis, a bacterium harboring a single cold shock protein encoding gene.* PloS one, 2014. **9**(3): p. e93289-e93289.

32. Wiegeshoff, F. and M.A. Marahiel, *Characterization of a mutation in the acetolactate synthase of Bacillus subtilis that causes a cold-sensitive phenotype.* FEMS Microbiology Letters, 2007. **272**(1): p. 30-34.

33. Hücker, S.M., et al., *Transcriptional and translational regulation by RNA thermometers, riboswitches and the sRNA DsrA in Escherichia coli O157:H7 Sakai under combined cold and osmotic stress adaptation.* FEMS Microbiology Letters, 2016. **364**(2).

34. Carty, S.M., K.R. Sreekumar, and C.R. Raetz, *Effect of Cold Shock on Lipid A Biosynthesis inEscherichia coli INDUCTION AT 12° C OF AN ACYLTRANSFERASE SPECIFIC FOR PALMITOLEOYL-ACYL CARRIER PROTEIN.* Journal of Biological Chemistry, 1999. **274**(14): p. 9677-9685.

35. Vorachek-Warren, M.K., et al., *An Escherichia coli mutant lacking the cold shock-induced palmitoleoyltransferase of lipid A biosynthesis: absence of unsaturated acyl chains and antibiotic hypersensitivity at 12 degrees C.* J Biol Chem, 2002. **277**(16): p. 14186-93.

36. van der Ley, P., et al., *Modification of lipid A biosynthesis in Neisseria meningitidis lpxL mutants: influence on lipopolysaccharide structure, toxicity, and adjuvant activity.* Infection and immunity, 2001. **69**(10): p. 5981-5990.

37. Han, Y., et al., *DNA microarray analysis of the heat- and cold-shock stimulons in Yersinia pestis.* Microbes and Infection, 2005. **7**(3): p. 335-348.

38. Galindo, C.L., et al., *Comparative Global Gene Expression Profiles of Wild-Type Yersinia pestis CO92 and Its Braun Lipoprotein Mutant at Flea and Human Body Temperatures.* Comp Funct Genomics, 2010: p. 342168.

39. Wang, P., A. Kuhn, and R.E. Dalbey, *Global change of gene expression and cell physiology in YidC-depleted Escherichia coli.* Journal of bacteriology, 2010. **192**(8): p. 2193-2209.

40. Belosludtsev, K., et al., *Interaction of Phospholipase A of the E. coli Outer Membrane with the Inhibitors of Eucaryotic Phospholipases A(2) and Their Effect on the Ca2+-Induced Permeabilization of the Bacterial Membrane.* The Journal of membrane biology, 2014. **247**.

41. Dekker, N., *Outer-membrane phospholipase A: known structure, unknown biological function.* Molecular Microbiology, 2000. **35**(4): p. 711-717.
